# Loss of full-length DNA replication regulator Rif1 in two-cell embryos is associated with zygotic transcriptional activation

**DOI:** 10.1016/j.jbc.2021.101367

**Published:** 2021-11-01

**Authors:** Naoko Yoshizawa-Sugata, Satoshi Yamazaki, Kaoru Mita-Yoshida, Tomio Ono, Yasumasa Nishito, Hisao Masai

**Affiliations:** 1 Genome Dynamics Project, Tokyo Metropolitan Institute of Medical Science, Tokyo, Japan; 2 Center for Basic Technology Research, Tokyo Metropolitan Institute of Medical Science, Tokyo, Japan

**Keywords:** 2C, two-cell (embryo), 4-OHT, 4-hydroxytamoxifen, Dox, doxycycline, ERV, endogenous retrovirus, ES, embryonic stem, Hpf, hours post fertilization, IDR, intrinsic disordered region, IVF, *in vitro* fertilization, KD, knockdown, KO, knockout, RT, room temperature

## Abstract

Rif1 regulates DNA replication timing and double-strand break repair, and its depletion induces transcriptional bursting of two-cell (2C) zygote-specific genes in mouse ES cells. However, how Rif1 regulates zygotic transcription is unclear. We show here that Rif1 depletion promotes the formation of a unique *Zscan4* enhancer structure harboring both histone H3 lysine 27 acetylation (H3K27ac) and moderate levels of silencing chromatin mark H3K9me3. Curiously, another enhancer mark H3K4me1 is missing, whereas DNA methylation is still maintained in the structure, which spreads across gene bodies and neighboring regions within the *Zscan4* gene cluster. We also found by function analyses of Rif1 domains in ES cells that ectopic expression of Rif1 lacking N-terminal domain results in upregulation of 2C transcripts. This appears to be caused by dominant negative inhibition of endogenous Rif1 protein localization at the nuclear periphery through formation of hetero-oligomers between the N-terminally truncated and endogenous forms. Strikingly, in murine 2C embryos, most of Rif1-derived polypeptides are expressed as truncated forms in soluble nuclear or cytosolic fraction and are likely nonfunctional. Toward the morula stage, the full-length form of Rif1 gradually increased. Our results suggest that the absence of the functional full-length Rif1 due to its instability or alternative splicing and potential inactivation of Rif1 through dominant inhibition by N-terminally truncated Rif1 polypeptides may be involved in 2C-specific transcription program.

Rif1, originally reported as a telomere-binding factor in yeasts, is evolutionally conserved and regulates chromosome transactions including DNA replication, DNA repair, transcriptional silencing, and telomere homeostasis. We and other groups previously reported that Rif1 plays a crucial role in organization of DNA replication timing during S phase in fission yeast (1, 2) and in mammals (3, 4).

Rif1 is abundantly present in preimplantation embryos, primordial germ cells, and embryonic pluripotent stem cells, while it is expressed at a low level in differentiated somatic cells (5). In mouse ES cells, multiple stem-cell-specific transcription factors are strongly expressed to maintain the core circuitry of the pluripotent state, but the expression levels of specific factors such as Nanog and Rex1 fluctuate in individual cells, resulting in heterogenous populations with different levels of each protein (6, 7). *Zscan4* (zinc finger and SCAN domain containing 4), previously identified as a gene expressed in late 2-cell zygote *in vivo*, has been also reported to be activated in ES cells in transient and sporadic manner in a rare population (1 to several %; (8)). Notably, *Zscan4* expression is accompanied by upregulation of a group of genes including *Tcstv1* and *Usp17l*, endogenous retrovirus (ERV) and noncoding RNA of major satellite, all of which is specifically transcribed in embryos at two-cell (2C) stage (9). This cellular state is often called “2C-like state” (10), but we will refer to it as “Zscan4-positive state” in this study. Many of the 2C genes exist as gene clusters that consist of multiple homologous gene family members, and most of them are upregulated simultaneously. Artificial induction of 2C genes was first reported in cells depleted of Kdm1a, a chromatin-binding protein with histone demethylation activity (11). Following this discovery, other factors were also shown to suppress 2C genes expression (12, 13, 14, 15, 16, 17). Rif1 is among them and is known to inhibit *Zscan4* and ERVs expression possibly through the maintenance of histone H3 lysine 9 trimethylation (H3K9me3), H3K27 trimethylation (H3K27me3), and DNA methylation (18, 19), although changes in other epigenetic marks and its causality of transcription remain unclear. In addition, the loss of Rif1 strongly induces 2C gene transcripts in ES cells, but not in other cell lines (3, 4). Studies on the roles of Rif1 in regulation of replication timing led to the notion that Rif1 regulates higher-order chromatin structure, since loss of Rif1 changes local chromatin interaction (20). Thus, the cell-specific chromatin structures may be linked to Rif1’s role in regulation of transcription, although no direct evidence for this idea has been presented.

Mammalian Rif1 protein has two major conserved domains (21); N-terminal HEAT-repeat, widely conserved in all the orthologs including yeast and fruit fly, is required for Rif1 foci formation caused by DNA damage and MAD2L2 recruitment in 53BP1-dependent manner (22, 23), whereas the C-terminal conserved region is required for interaction with BLM/RMI1/RMI2 complex, DNA binding, and self-oligomerization (24). Our biochemical studies have revealed that both Rif1 N-terminal 1151 aa and C-terminal 299 aa domains independently form oligomers, tetramer or octamer, and dimer or dodecamer, respectively, and bind to G-quadruplex (25). On the other hand, both N-terminal and C-terminal domains are required for Rif1-mediated suppression of Brca1 foci and for stimulation of nonhomologous end-joining DNA repair, suggesting that multimerization through both N-terminal and C-terminal regions may be required for this process. For the suppression of *MERVL* in ES cells, N-terminal HEAT-repeat of Rif1 is required (19), but detailed domain analyses including the effects of overexpression have not been addressed so far.

Here, we examined the chromatin structure and epigenetic states of *Zscan4* loci and unexpectedly found that silent marks including DNA methylation and H3K9me3 are mostly maintained even in the absence of Rif1, whereas H3K27ac is markedly upregulated as a single active histone mark. Although the increase of hyper acetylation of H3K27 correlates with Zscan4-positive state, this modification is not the sole cause for derepression. Surprisingly, the truncated forms of Rif1 lacking N-terminal HEAT repeat induced Zscan4-positive state in ES cells when transiently expressed, possibly through perturbation of multimer formation and/or dislocalization of endogenous Rif1. Finally, the analyses of endogenous Rif1 protein *in vivo* by immunostaining and immunoblotting suggested that the reduced amount of full-length Rif1 and the dislocalized truncated polypeptides lead to the loss of function of Rif1 in oocytes and embryos, which is linked to the derepression of 2C-specific transcripts.

## Results

### Rif1 is dispensable for the maintenance of undifferentiated states

**Figure 1:**
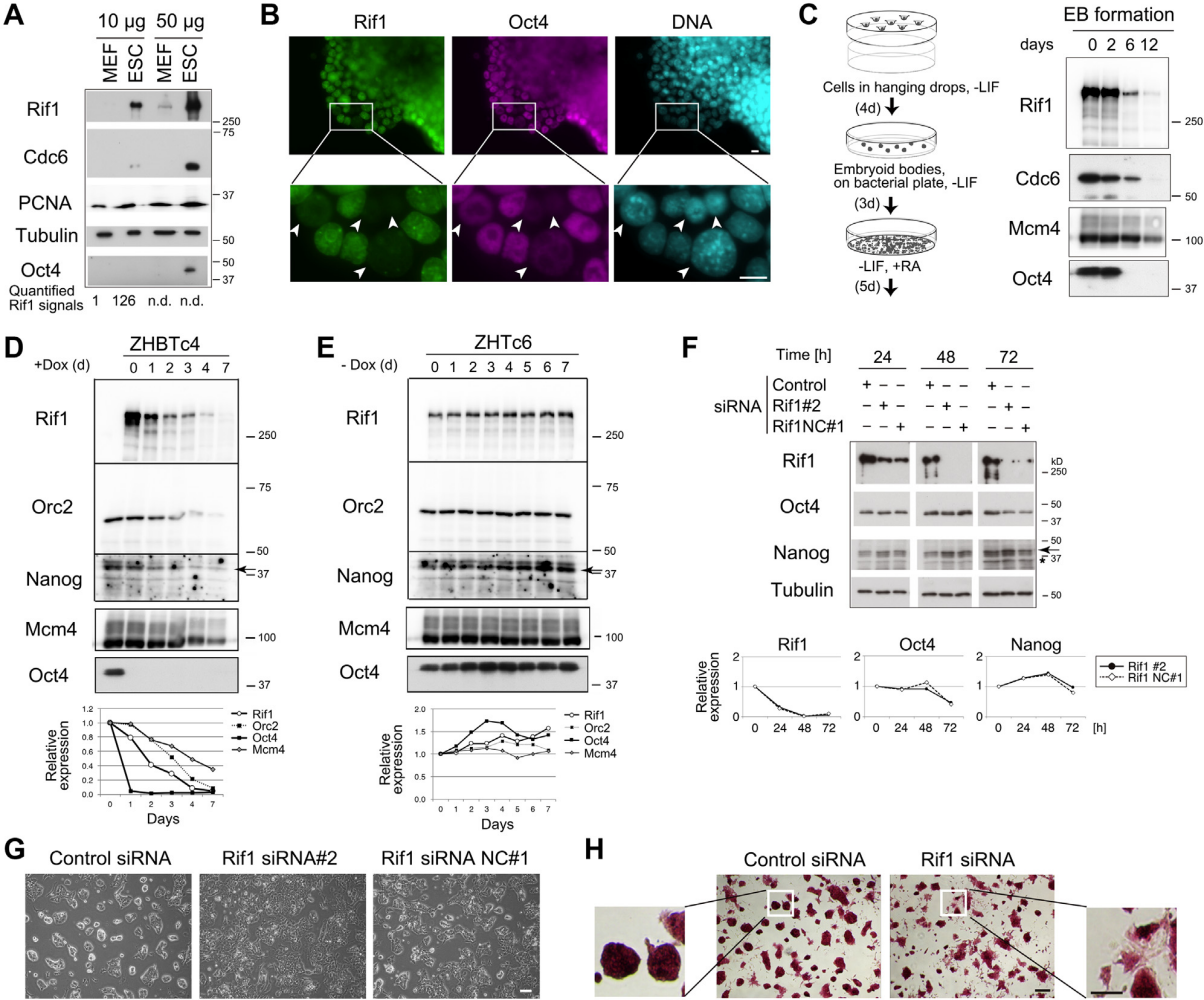
**Rif1 is expressed in undifferentiated mouse ES cells but is not essential for maintenance of undifferentiated state.***A*, immunoblot analyses of Rif1 and other proteins in D3 mouse ES cells or mouse embryonic fibroblast (MEF) cells. Rif1 signal intensities were quantified for lanes 1 and 2 (*bottom*). *B*, immunofluorescence images of D3 cells. The cells overgrown for 3 days were fixed and subjected to immunofluorescence staining using anti-Rif1 UCRIII (*left panels*) or anti-Oct4 (*middle panels*) antibodies. DNA was costained with DAPI (*right panels*). Enlarged images of marked areas are shown in the *lower panels*. *Arrowheads* indicate the cells that have undergone spontaneous differentiation and exhibit reduced Rif1 and Oct4 levels. Bars, 10 μm. *C*, immunoblots of E14tg2a cells after induced differentiation by embryoid body formation. *D*, immunoblots of ZHBTc4 cells after depletion of Oct4 gene. *E*, immunoblots of ZHTc6 cells after overexpression of Oct4 gene. In (*C*–*E*), the whole cell extracts (10 μg proteins) were analyzed by indicated antibodies. *F*, immunoblot analysis of mouse E14tg2a cells transfected with siRNA targeting Rif1. The whole cell extracts (10 μg proteins) were analyzed by indicated antibodies. The relative expression levels of Rif1, Oct4, and Nanog proteins are shown in the bottom graphs. The levels of control cells at 0 h were set as 1. *G*, the phase-contrast images at 48 h after knockdown in (*F*). Bar, 100 μm. *H*, alkali phosphatase-staining images. The cells transfected with siRNA Rif1 NC#1 in (*F*) were fixed at 96 h and subjected to staining. Bars, 200 μm for center panels, or 100 μm for side magnification panels. Two or more independent experiments were performed for each analysis.

The expression levels of DNA replication-regulatory proteins vary widely among cell types. In pluripotent ES cells, prereplicative complex components, Cdc6 and Orc2, are abundantly present compared with embryonic fibroblast cells, while DNA replication sliding clamp PCNA is at similar levels in both cells (Fig. 1*A*). Rif1 is more highly expressed in ES cells (>100-fold) than in fibroblasts, as previously reported (Fig. 1*A*; (5, 18)). Rif1 expression is rapidly reduced during spontaneous differentiation (Fig. 1*B*) or induced differentiation by embryoid body formation, concomitant with the loss of Oct4 expression (Fig. 1*C*; (5)). Since Rif1 is one of the major targets of several pluripotent transcription factors including Oct4, Sox2, Nanog, and Dax1/Nr0b1 (26, 27), in this study, we depleted Oct4 (encoded by *Pou5f1* gene) in ZHBTc4 cells (28). As expected, depletion of Oct4 resulted in an abrupt decrease of Rif1 in 1 day (Fig. 1*D*) and its overexpression increased Rif1 protein by 1.7-fold in 3 days (Fig. 1*E*), showing that Rif1 expression is correlated with the Oct4 protein level and undifferentiated states.

Meanwhile, the effects of Rif1 depletion on undifferentiated states of ES cells have been controversial (20, 26, 29), thus we depleted Rif1 by siRNA in ES cells (Fig. 1*F*). As a result, most population of cells retained alkali phosphatase activities with minor changes in ES cell-like morphology, showing that Rif1 knockdown (KD) has marginal effects on the maintenance of undifferentiated states (Fig. 1, *G* and *H*). The mRNA and protein levels of Oct4 and Nanog along with other pluripotent markers were not markedly reduced by Rif1 KD (Figs. 1*F* and S1*A*). To achieve complete depletion of Rif1, we generated Rif1 conditional knockout (KO) ES cell lines (*mRif1*^*flox/flox*^ *CreERT2*) and confirmed that the levels of pluripotent marker transcripts were not reduced by Rif1 KO, except for moderate upregulation of Nanog (approximately 1.8-fold) (Fig. S1*B*). In addition, the major differentiation markers were unchanged by Rif1 KO, although some genes were dysregulated (Fig. S1*B*). On the basis of these data, we have concluded that Rif1 is dispensable for maintenance of undifferentiated state of ES cells.

### Rif1 inhibits emergence of 2C gene-upregulated states of ES cells

**Figure 2:**
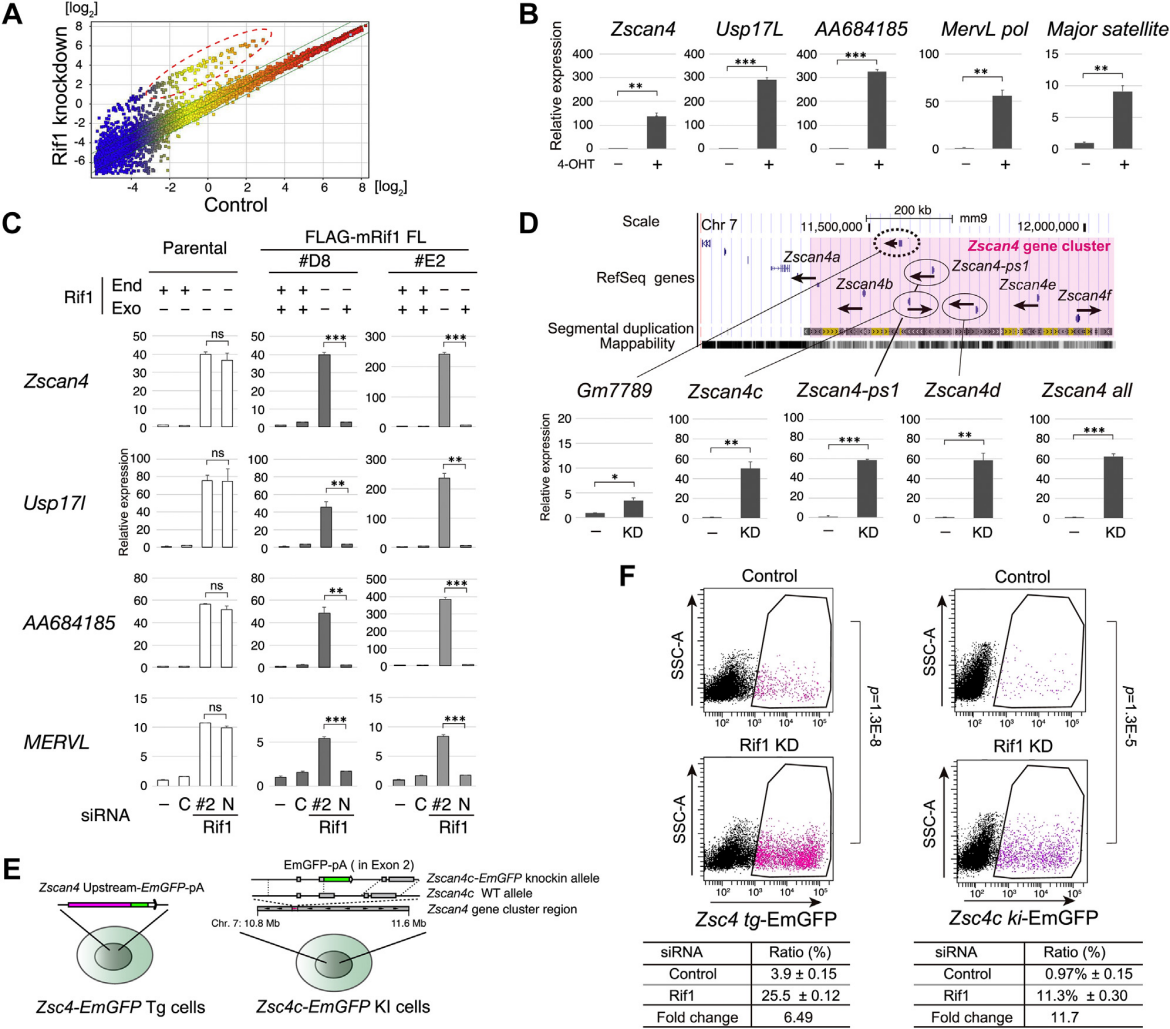
**Depletion of Rif1 derepresses 2C-specific transcripts: more efficient activation of reporters in the endogenous *Zscan4c* locus than those randomly integrated.***A*, the scatter plot of transcriptome analysis of Rif1 knockdown cells (Y axis) and control cells (X axis) at 72 h. One out of two independent experiments was shown. *B*, qRT-PCR of Rif1 knockout cells. The relative transcript levels of indicated genes in *Rif1*^*f/f*^*CreERT2* cells treated with 4-hydroxytamoxifen (4-OHT) for 48 h (+) or untreated (−) are shown. *C*, complementation assay. Two lines (#D8 and #E2) of stable transformant cells expressing FLAG-tagged mRif1 were transfected twice with siRNA against both endogenous and exogenous Rif1 (siRNA#2; #2) or that against only endogenous Rif1 (siRNA NC#1; N), or that with random sequence (Control; C), or untreated (−). The cells were harvested at 48 h and the relative expression levels of indicated transcripts were determined by qRT-PCR. *D*, genome structure of the *Zscan4* gene cluster region and the deregulated genes upon Rif1 knockdown (KD). The regions containing the left end (650 kb) of the *Zscan4* gene cluster are shown in UCSC genome browser map of mouse genome (NCBI37/mm9) (*top*). The transcript levels of indicated genes in Rif1 KD relative to control (−) at 72 h are shown (*bottom*). In (*B*–*D*), the means of technical triplicates in one out of two independent experiments were shown and analyzed by using unpaired two-tailed Student’s *t* test. ∗, *p* < 0.05; ∗∗, *p* < 0.01; ∗∗∗ *p* < 0.001. Bars, SDs. *E*, Zscan4-reporter cells used in this study. The cells harboring the *Zscan4* promoter (2.6 kb)-fused *EmGFP* transgene (*Zsc4-EmGFP* Tg) generated by random integration (*left*) or the *EmGFP* reporter knocked in the endogenous *Zscan4c* gene locus (*Zsc4-EmGFP* KI; *right*) were previously reported (9, 31). *F*, derepression of Zscan4 in *Zsc4-EmGFP* Tg (*left*) and *Zsc4-EmGFP* KI cells (*right*). The cells were transfected with Rif1 siRNA #2 twice and harvested at 48 h and analyzed by flow cytometry. GFP-positive cells in boxed region are shown by magenta and marked in *dot* plots. The representative data out of two independent experiments were shown. The means of biological triplicates and the fold changes are shown in the *bottom* tables. *p*-values of unpaired two-tailed Student’s *t* test were shown on the right side of the plots.

To evaluate the effects of mRif1 depletion, we conducted gene expression analysis using whole mouse genome microarray. While pluripotent markers were unchanged, a fraction of genes were upregulated in Rif1 KD cells (>log_2_2-fold, 1511 entities; Fig. 2*A*), and 43 out of the top 100 transcripts belong to a group of family genes that are specifically expressed in embryos at 2C stage and are sporadically and periodically activated in a limited population in ES cells (8) (Table 1). This is consistent with a previous report (18). Most of them belong to gene clusters ranging in size from 15 kb to 2 Mb consisting of highly homologous gene family members. The transcript levels of these 2C genes were strikingly elevated in Rif1 knockdown cells (14–30-fold; Table 1). Recently, other chromatin-binding proteins have been reported to repress the 2C-specific transcripts of ES cells; these include Kdm1a (11), Polycomb group proteins (*e.g.*, RYBP (12), Pcgf6 (13), MAX (14)), GSK3 (15), Dax1/Nr0b1 (16) and Chaf1 (chromatin assembly factor-1) (17). Complete deletion of Rif1 in *mRif1*^*flox/flox*^ *CreERT2* cells resulted in even more striking increase of 2C genes (100–300-fold, Fig. 2*B*) along with moderate dysregulation of differentiation-related genes (<5-fold; Fig. S1*B*). In addition, the transcripts of endogenous retrovirus *MERVL* and noncoding RNA of major satellite repeats were also extensively induced in Rif1 KO ES cells, as seen in 2C embryos and naturally occurring Zscan4-positive state cells (Fig. 2*B*). We cloned full-length Rif1 cDNA and established stable transformant cell lines expressing ectopic Rif1 for complementation assays. Rif1 siRNA-mediated derepression of *Zscan4, Usl17l, AA684185,* and *MERVL* was rescued by exogenous Rif1 expression in two independent stable clones (Fig. 2*C*). Taken together, our results firmly establish that Rif1 inhibits the dysregulated transcription of 2C genes in ES cells.

**Table 1:** Highly upregulated transcripts in Rif1 knockdown cells are in gene clusters and specifically expressed in two-cell zygotes

Genes		Chr	Cluster size (kb)	Gene # in cluster	Expression level	Fold change	Entities in top 100
Upregulated genes in Rif1 knockdown	*Tcstv1*	13	425	11	59.8	15	5
	*Gm4340*	10	54	7	56.3	14	1
	*Zscan4*	7	880	9	51.4	17	7
	*Gm2022*	12	815	17	17.0	21	14
	*Gm4303*	10	91	12	11.6	20	3
	*Gm13043*	4	847	8	10.7	18	2
	*Usp17l*	7	1610	5	8.2	22	3
	*AA684185*	18	15	7	8.1	14	2
	*Gm7682*	5	2427	46	4.9	21	3
	*Gm428*	4	82	6	4.3	30	2
	*Tmem92*	11	105	7	3.7	23	1
Unaffected	*Zfp42*	8	-	1	53.6	1.0	0
	*Gfap*	11	-	1	0.0022	1.1	0

1511 genes were upregulated in Rif1 knockdown cells at 48 h with fold change log_2_2< compared with control siRNA treated cells. Top 100 entities in the expression levels were identified by BLAST search, and among them, 43 entities grouped into 11 gene families that are transcribed specifically at the two-cell zygote stage (10) were listed. The genomic structures (cluster size and numbers of gene family members) were confirmed by mouse reference genome NCBI37/mm9 assembly. The median of the expression levels and fold changes of multiple microarray data of family genes, except for *Gm4340* and *Tmem92*, are presented. For comparison, data of pluripotent gene *Zfp42* and neural marker *Gfap* were shown. The median of all 27,732 entities was 0.070.

### Rif1 suppresses the transcription of the *Zscan4* gene cluster through the individual promoters as well as through other unknown genetic elements

Zscan4 is required for maintenance of telomere functions in ES cells and in embryos and is essential for development (9). Nine *Zscan4* gene family members are dispersed in the *Zscan4* gene cluster of 880 kb in length on the chromosome 7 (Figs. 2*D* and S1*C*) with the following three features; (1) All of the nine genes are transcribed in ES cells and possibly express proteins (15, 30); (2) Sequences of at least 40 kb containing the coding and surrounding noncoding regions are highly homologous, resulting in segmental multiplication of sequences for submegabases (Fig. S1*C*); (3) Various endogenous retrotransposable elements and their remnants are frequently present within this gene cluster. (Fig. S1*C*; (11)). To investigate mode of gene regulation by Rif1, we selected the *Zscan4* gene cluster as a model for Rif1-regualted gene clusters that share similar features. We first analyzed the expression of *Zscan4* gene family members by qRT-PCR using single member-specific or common primers and found that all *Zscan4* gene family members tested were derepressed (50–60-fold) by Rif1 knockdown (Fig. 2*D*). In contrast, the transcripts for the non-*Zscan4* family gene (*Gm7789*) localized between *Zscan4b* and *Zscan4c* were moderately upregulated by Rif1 depletion (3.4-fold). This indicates that Rif1 depletion may suppress the transcription of the whole *Zscan4* gene cluster region but that it affects the *Zscan4* promoter more profoundly. Next, we asked whether the regulatory sequence of each gene is sufficient for repression of the *Zscan4* gene by Rif1. To answer this, we used 2 cell lines; *Zscan4-Emerald GFP (EmGFP)* transgene MC1 cells carry *EmGFP* fused to the 2.6 kb upstream sequence of the *Zscan4c* gene integrated in its genome (hereafter *Zsc4-EmGFP* Tg cells; (9)), whereas *Zscan4c-EmGFP* knock-in TA1 mouse ES cells (*Zsc4c-EmGFP* KI cells; (31)) carry *EmGFP* at the endogenous *Zscan4c* gene loci (Fig. 2*E*). EmGFP-positive population increased by 6.5-fold in *Zsc4-EmGFP* Tg cells, as was reported previously (Fig. 2*F*, left; (18)), while EmGFP-positive population increased by 11.7-fold in *Zsc4c-EmGFP* KI cells by Rif1 KD (Fig. 2*F*, right). Not only EmGFP-positive (+) population but also the fluorescent signal intensities increased by Rif1 depletion in both reporter cells (Fig. S2*A*), suggesting that both the probability and extent of promoter regulation are affected (Fig. S2, *B*–*F*). The results indicate that the upstream regulatory region of *Zscan4* contains information sufficient for repression by Rif1, but some other genetic elements present in the gene cluster may also contribute to the repression.

### DNA methylation of *Zscan4* gene loci is reduced but still maintained

**Figure 3:**
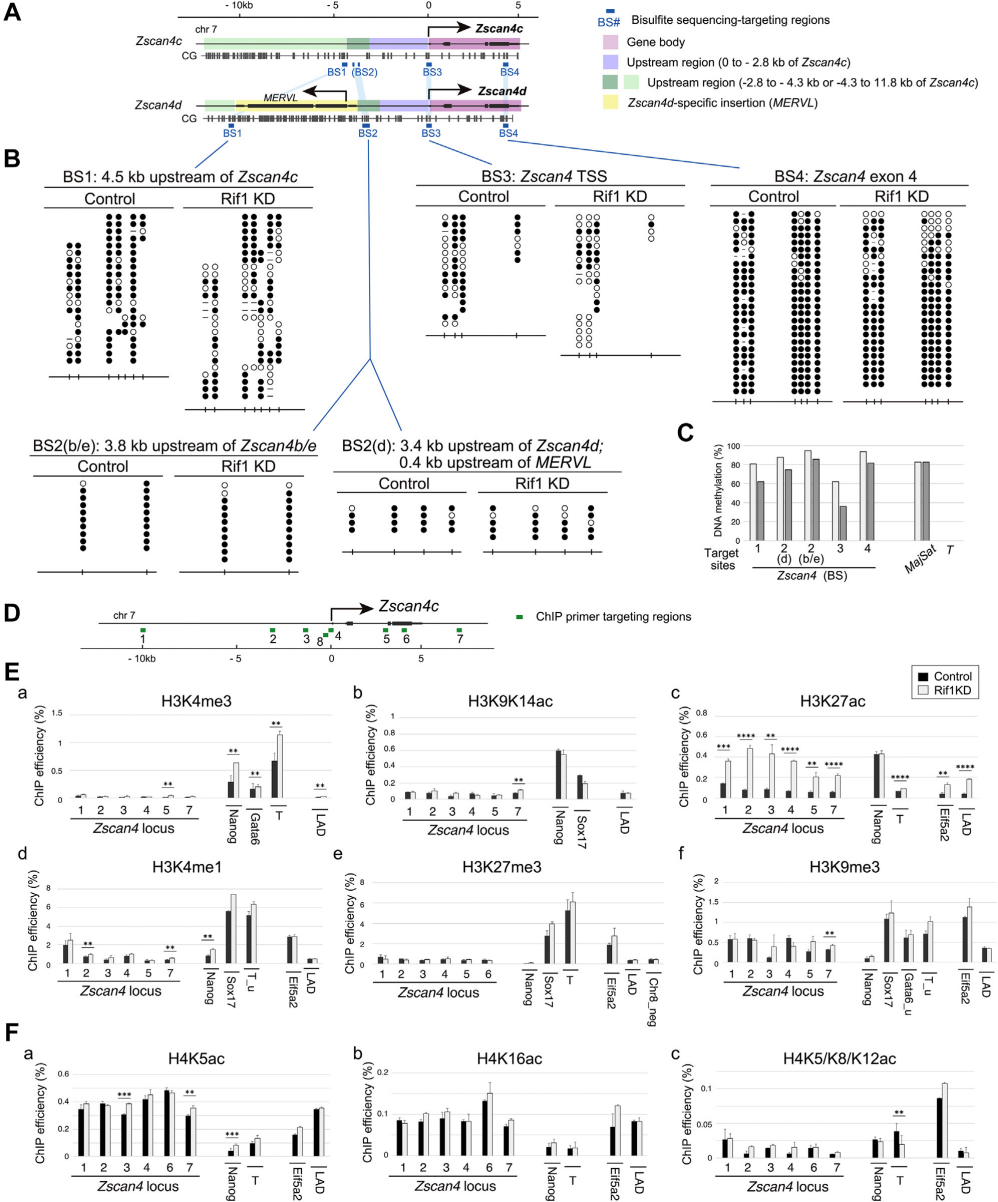
**DNA methylation and histone modification analysis of the *Zscan4* gene loci in Rif1-depleted cells.***A*, map of the *Zscan4* loci with genomic structure, CpG sites and regions targeted for bisulfite sequencing analyses. The *Zscan4c* and *Zscan4d* gene loci are shown as examples and regions with sequence similarity are shown by *color* codes. Note that the BS2 target segments of only *Zscan4b*, *d,* and *e* have CpG sites. *B*, bisulfite sequencing analysis of indicated targeting regions. For BS2, the results of two CpG sites from *Zscan4b* or *Zscan4e* and four CpG sites from *Zscan4d* are shown, out of total 29 clones. *Open circles* and *filled circles*, unmethylated or methylated CpG, respectively; *Open spaces*, no CpGs in reference sequences of corresponding family genes; bars, no CpGs probably due to cell line-specific mutations or single nucleotide polymorphisms in the mouse strain. *C*, summary of the results in (*B*). For BS1, methylation rate calculated by sequence polymorphisms occurrence is shown (See Experimental procedures for details). Two independent experiments were done for (*B*). *D*, map of genomic structure of the *Zscan4c* gene (shown as a representative of the *Zscan4* family genes) and target regions of ChIP primers. *E* and *F*, ChIP-qPCR analysis of modification of histone H3 (*E*) or H4 (*F*). The means of triplicated data are plotted. *Black*, control; *white*, Rif1 knockdown. Control target loci are as follows; *Nanog*, a pluripotent marker gene; *T*, *Sox17* and *Gata6*, differentiation regulatory genes; *Eif5a2*, a moderately silent region with H3K9me3 mark; LAD, a LaminB-binding site; Chr8_neg, H3K27me3-negative region. For *T*, the upstream regions were analyzed by two primer sets; T for targeting promoter region or T_u for enhancer region. Bars, SDs. For (*E* and *F*), a minimum of two independent experiments were performed. The means of technical triplicates of representative data were shown and analyzed by using unpaired two-tailed Student’s *t* test. ∗∗, *p* < 10^−2^. ∗∗∗, *p* < 10^−3^. ∗∗∗∗, *p* < 10^−4^. No marks, not significant. Bars, SDs.

*MERVL*, a 2C-specific endogenous retrovirus (ERV) transcript, was also extensively upregulated in Rif1 depleted cells (54.9-fold; Fig. 2*B*) as in intrinsic Zscan4-positive cells. It has been suggested that DNA methylation regulates certain ERV expression (32), thus we analyzed DNA methylation of MspI site (CCGG) in the 3.3 kb upstream region of the *Zscan4e* gene by HpaII assay (Fig. S3*A*). We found similar levels of methylation in both untreated and Rif1-depleted ES cells, although sensitivity to HpaII (incapable of cleaving CpG methylated CCGG) slightly increased in the Rif1 knockdown cells (Fig. S3*A*). We further conducted bisulfite sequencing of four targeting sites in the *Zscan4* gene loci; two upstream regions (BS1 and BS2), transcription start site (TSS; BS3), and gene body (BS4) (Fig. 3*A*). The upstream regions and gene body are highly methylated (over 88%; Fig. 3, *B* and *C*) as previously reported (33), while TSS is less methylated (63%; Fig. 3, *B* and *C*). When depleted of Rif1, DNA methylation was moderately reduced at BS1 (4.5 kb upstream of *Zscan4c*) from 81% to 62% [×0.77] or at BS3 (TSS) from 63% to 35% [×0.57], but less affected at BS4 (gene body; from 94% to 82% [×0.87]) (Fig. 3, *B* and *C*). Next, we examined BS2, targeting the 3.8 kb upstream of *Zscan4b* or *Zscan4e*, and the 3.5 kb upstream of the *Zscan4d* gene where *MERVL* is inserted in head-to-head direction, to confirm whether the adjacent *MERVL* influences the DNA methylation of the *Zscan4d* locus. To do this, analyzed sequences were identified either as *Zscan4b/e*-derived or *Zscan4d*-derived on the basis of single nucleotide polymorphism information in NCBI database. Although the *MERVL* near *Zscan4d* was actively transcribed in Rif1 depleted cells (19), DNA methylation in the *Zscan4d* locus only slightly decreased (88% to 75% [×0.85], similar to the *Zscan4b/e* loci (95% to 86% [×0.91]) (Fig. 3, *B* and *C*). We concluded that genomic DNA methylation of the *Zscan4* gene cluster region is largely maintained at a high level in mRif1-depleted ES cells, regardless of the presence or absence of adjacent *MERVL*.

### Rif1 depletion is associated with increased H3K27 acetylation but with no other histone H3 modifications in the *Zscan4* gene loci

**Figure 4:**
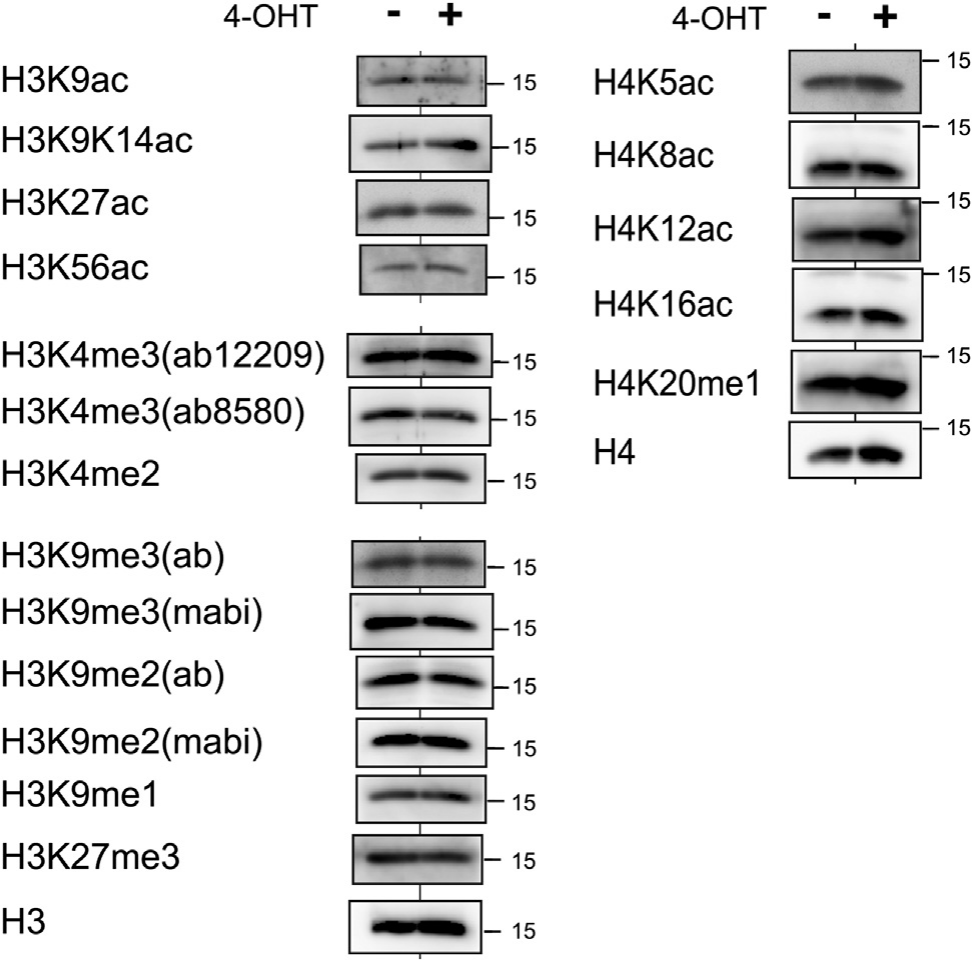
**Histone modification on the whole chromatin.** Histone-enriched fractions were prepared by acid-extraction methods from *Rif1*^*f/f*^*CreERT2* mouse ES cells incubated with 4-OHT for 48 h (+) or untreated (−). 0.1 μg, 0.3 μg, or 3 μg proteins were used for immunoblot analysis for pan H3 and H4, H3K9K14ac and H3K56ac, or other modified histones, respectively. The representative blots from triplicate or duplicate experiments are shown. Bars indicate 15 kD markers.

Apart from DNA methylation, ERVs are known to be silenced by histone modification, such as histone H3 lysine 9 methylation (H3K9me2/3; (34, 35)), H3 lysine 27 trimethylation (H3K27me3; (36)), or removal of active histone marks (11). Since typical 2C genes clusters consist of multiple highly homologous sequences and accurate mapping of chromatin immunoprecipitation-sequencing (ChIP-seq) reads is difficult, we conducted ChIP-qPCR analysis to quantify histone modification using primers targeted to the *Zscan4* gene region (Fig. 3*D*). Active marks, H3K4me3 and H3K9/K14ac, were low in untreated ES cells and unexpectedly, did not change by Rif1 knockdown (Fig. 3*E*, a and b). In contrast, H3K27 acetylation (H3K27ac), generally known as an enhancer mark, significantly increased in the all tested sites including the ones at TSS, in the gene bodies and the surrounding regions (up to 10 kb upstream and to 1.9 kb downstream of *Zscan4*; Fig. 3*E* c). The broadly spreading H3K27ac mark is reminiscent of “super-enhancers,” which is characterized by colocalization of another enhancer mark H3K4me1 and binding of pluripotent transcription factors and mediators (37). However, H3K4me1 marks were not notable in this region both in untreated and in Rif1-depleted cells (Fig. 3*E* d), unlike in super-enhancers. We then asked whether the prominent increase of H3K27ac was caused by removal of H3K27 silent marks, but the *Zscan4* loci had no H3K27me3 mark in untreated cells, and Rif1 depletion had no effects (Fig. 3*E* e). Furthermore, another silent mark H3K9me3 was moderately present in untreated cells and was reduced in Rif1 knockdown cells (Fig. 3*E* f) but was clearly remained (at the levels higher than in *Nanog* locus and lower than in differentiation marker locus of *Sox17* or *T*). Similar results were obtained also in Rif1 KO ES cells derived from the different mouse strain (Fig. S3*B*). Other marks of histone H4 including H4K5ac, H4K8ac, H4K5/K8/K12ac, and H4K20me1 were also unchanged at most of the locations examined, except for moderate increase of H4K16ac in Rif1 knockout cells (Figs. 3*F* and S3*C*). Importantly, we found no significant changes (>2-fold) of histone modification in biochemically fractionated samples (Fig. 4), indicating that no global changes in posttranslational modification of histone H3 and H4. From this result, we conclude that increase of broad H3K27ac enhancer marks might be restricted to the regions surrounding *Zscan4,* and presumably to other 2C genes, or in a limited population of Rif1-depleted cells, while H3K9me3 mark is mostly maintained.

### H3K27 hyperacetylation in heterochromatin region increases by Rif1 depletion but is not a prerequisite for *Zscan4* activation

**Figure 5:**
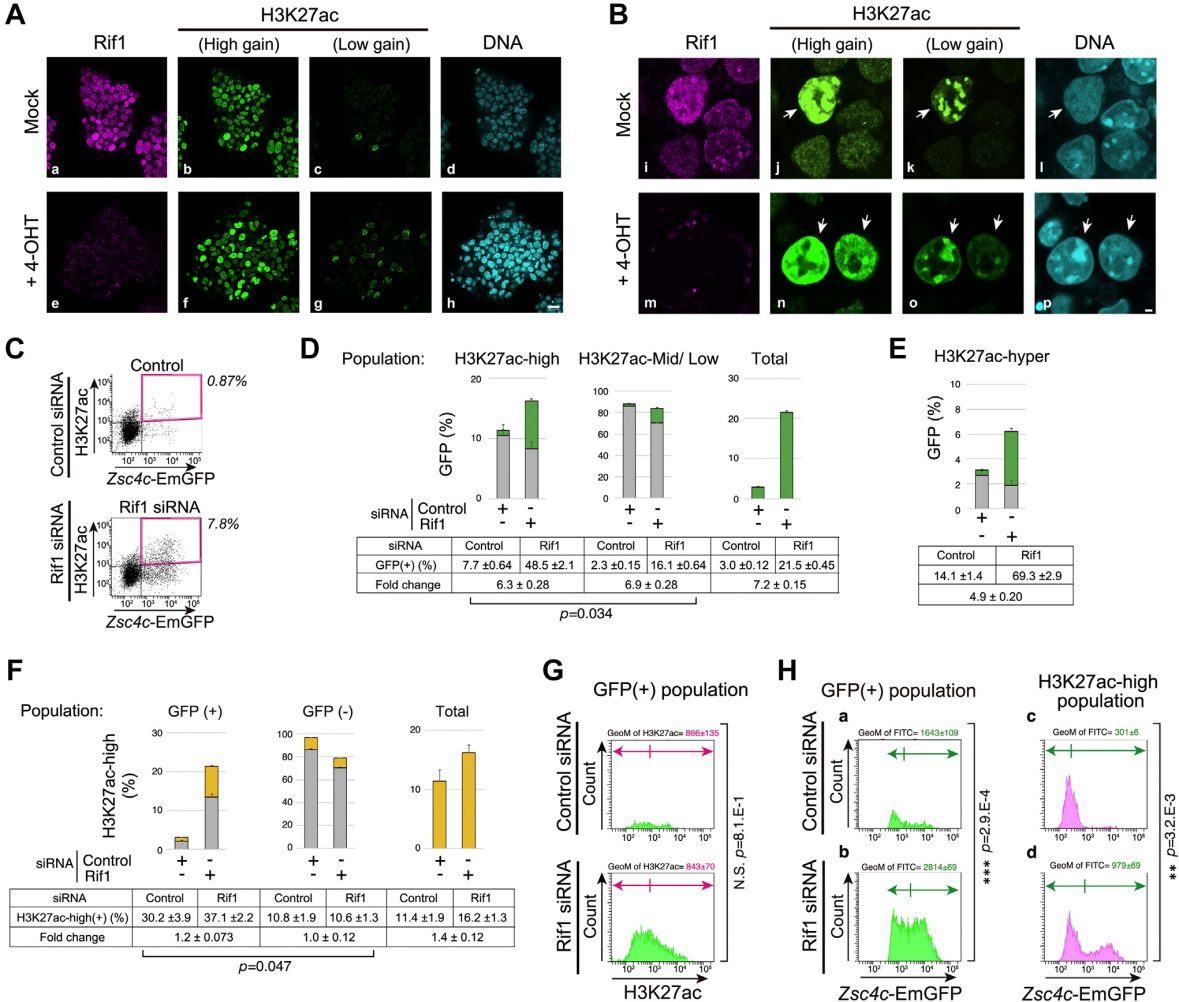
**High H3K27 acetylation is associated with Zscan4-positive state but is not a sole factor for Zscan4 deregulation.***A* and *B*, *Rif1*^*f/f*^*CreERT2* cells treated with 4-OHT or mock treated (Mock) were cultured for 72 h, fixed and subjected to immunostaining using anti-Rif1N and H3K27ac antibodies. DNA was costained with DAPI. Representative images acquired by fluorescent confocal microscopy at low (*A*) or high (*B*) magnification are shown. Bars, 20 μm (*A*) and 2 μm (*B*). *C*–*H*, *Zsc4c-EmGFP* KI cells transfected with Rif1 siRNA (siRNA#2) or control siRNA twice and harvested at 48 h. Fixed cells were subjected to immunostaining using anti-GFP and H3K27ac antibodies, and analyzed by flow cytometry. *C*, representative dot plots of GFP and H3K27ac are shown. *D*, GFP-positive fractions (*green*) in the regions of H3K27ac-high (*left*), H3K27ac-mid/low (*mid*) and the whole population (*right*) are indicated. *E*, GFP-positive fractions in the H3K27ac-hyper population (3% in untreated cells) are indicated. *F*, fractions of H3K27ac-high ratio (*orange*) in regions of GFP(+), and GFP(−), and in total population. In (*D*–*F*), the means of biological triplicates are shown in the tables. Bars, SD. Induction of GFP(+) ratio (*D*) or H3K27ac-high ratio (*F*) caused by Rif1 knockdown was compared between different populations (parentheses) by using two-tailed student’s *t* test. *p*-values shows only a small difference between two groups with different levels of H3K27ac or GFP, respectively. The selected regions in the FACS profiles are shown in Figure S3, *E*–*G*. *G*, GFP-positive populations are shown in the histograms of H3K27ac. *H*, GFP-positive (a and b) and H3K27ac-high (c and d) populations are shown in the GFP histograms. In (*G* and *H*), geometrical means (GeoM; at the *top* of each panel) along with its statistical differences (*p*-values) assessed by two-tailed student’s *t* test are shown at the *right side* of each pair. The selected regions in the FACS profiles in (*G*) and (*H*) are shown in Figure S3*H*. Two or more independent experiments with biological triplicates were performed for each analysis. N.S., no significance.

To monitor the changes in chromatin structure in single nuclei, we detected H3K27ac by indirect immunofluorescent microscopy in control and Rif1 KO cells. Although the overall signal intensities were unchanged (Fig. 5*A*, b and f), consistent with the results of the immunoblot analysis (Fig. 4, left, third panel), the cell population with high H3K27ac-staining increased in Rif1 KO cells compared with untreated cells (Fig. 5*A*, g and c, respectively). Normally, H3K27ac is localized at transcriptionally active euchromatin domains, but is temporarily and strongly localized in heterochromatin in spontaneously appearing Zscan4-positive and other 2C gene-positive cells (Z4 events; (33)). We also detected the bright patches of H3K27ac signals in a couple of percent of untreated cells without detectable DAPI-dense heterochromatin structure (chromocenters) (Fig. 5*B*, j–l; arrows). Dispersion or reorganization of DAPI-dense chromocenter is one of the characteristic features of Zscan4-positive state in ES cells (33), and Chaf1 depletion also results in loss of chromocenter (38) along with reactivation of *MERVL* (17). Interestingly, cells with high H3K27ac signals in bright patches were more frequently observed in Rif1 KO cells than control cells (Fig. 5*A* g and *B*, n and o), but chromocenters were mostly unaffected (Fig. 3*A* h and *B* p; arrows), suggesting that there might be some differences in heterochromatin structures in Zscan4-positive cells induced by depletion of Rif1 and Chaf1.

To examine whether elevated H3K27 acetylation is a determinant for derepression of *Zscan4* in Rif1-depleted cells, we analyzed GFP and H3K27ac signals by flow cytometry using *Zsc4c-EmGFP* KI cells. First, we fractionated cells into GFP-positive (+) and GFP-negative (−) cells, and H3K27ac-high and H3K27ac-mid/low (Figs. 5*C* and S3, *E* and *F*). In control, only 0.87 ± 0.07% of cells were GFP(+)/H3K27ac-high, and Rif1 depletion increased this fraction to 7.8 ± 0.26% (Fig. 5*C*, region marked by magenta). GFP(+) population in control cells of H3K27ac-high and H3K27ac-mid/low cells was 7.7% and 2.3%, respectively, and Rif1 knockdown upregulated GFP(+) ratio in both populations (49% and 16%, respectively; Fig. 5*D*), resulting in the increase of GFP(+) cells by similar ratios in H3K27-high and H3K27ac-mid/low cells (6.3 and 6.9-fold, respectively). Next, we gated H3K27ac-“hyper” population, constituting approximately 3% of the total cells, consistent with the estimation from the immunofluorescent images (Fig. 5*A* c). The GFP(+) ratio in this population was 14% in control, but increased to 70% in Rif1-depleted cells (4.9-fold; Fig. 5*E*). This indicates that Rif1 depletion increases the probability of the Zscan4-positive state in cells with H3K27ac-hyper mark, although Rif1 depletion-mediated enhancement of GFP(+) does not require high H3K27ac state since GFP(+) ratio is also increased in H3K27ac-mid/low populations (6.9-fold; Fig. 5*D*). Conversely, GFP(+) cells are more associated with H3K27ac-high mark than in GFP(−) cells (30% and 11%, respectively), although Rif1 KD did not significantly affect these ratios (37% and 11%, respectively; Fig. 5*F*). The geometric mean of H3K27ac of GFP(+) population was not significantly affected by Rif1 knockdown (Fig. 5*G*), in accordance with the fact that the whole-cell H3K27ac level is not affected by Rif1 depletion.

We next analyzed the GFP signal levels of selected populations. The *Zscan4c*-EmGFP signal intensities of GFP(+) population were much higher in Rif1-depleted *Zsc4c-EmGFP* KI cells than in control cells (*p* < 0.001; Fig. 5*H*, a and b) due to the increased number of cells with higher GFP levels (Fig. S2*F*). *Zscan4c*-GFP signals of H3K27-high populations in Rif1 knockdown cells were also higher (*p* < 0.01) than those in control cells (Fig. 5*H*, c and d). Interestingly, activation of *Zscan4c-*GFP(+) by Rif1 depletion occurred only in the limited fractions of H3K27-high population and resulted in two-phase distribution of extremely high GFP(+) and GFP(−) (Fig. 5*H* d). Consistent with this, fluorescent microscopic images have also indicated that only a fraction of GFP(+) cells are accompanied with high H3K27 mark and not all high H3K27ac cells are GFP-positive (Fig. S3*I*, arrowheads). In summary, global high H3K27 acetylation is strongly associated with derepression of *Zscan4* but is not a sole factor for *Zscan4* deregulation in Rif1-depleted cells.

### Overexpression of Rif1 deletion mutants containing C-terminal region increases Zscan4-positive state

**Figure 6:**
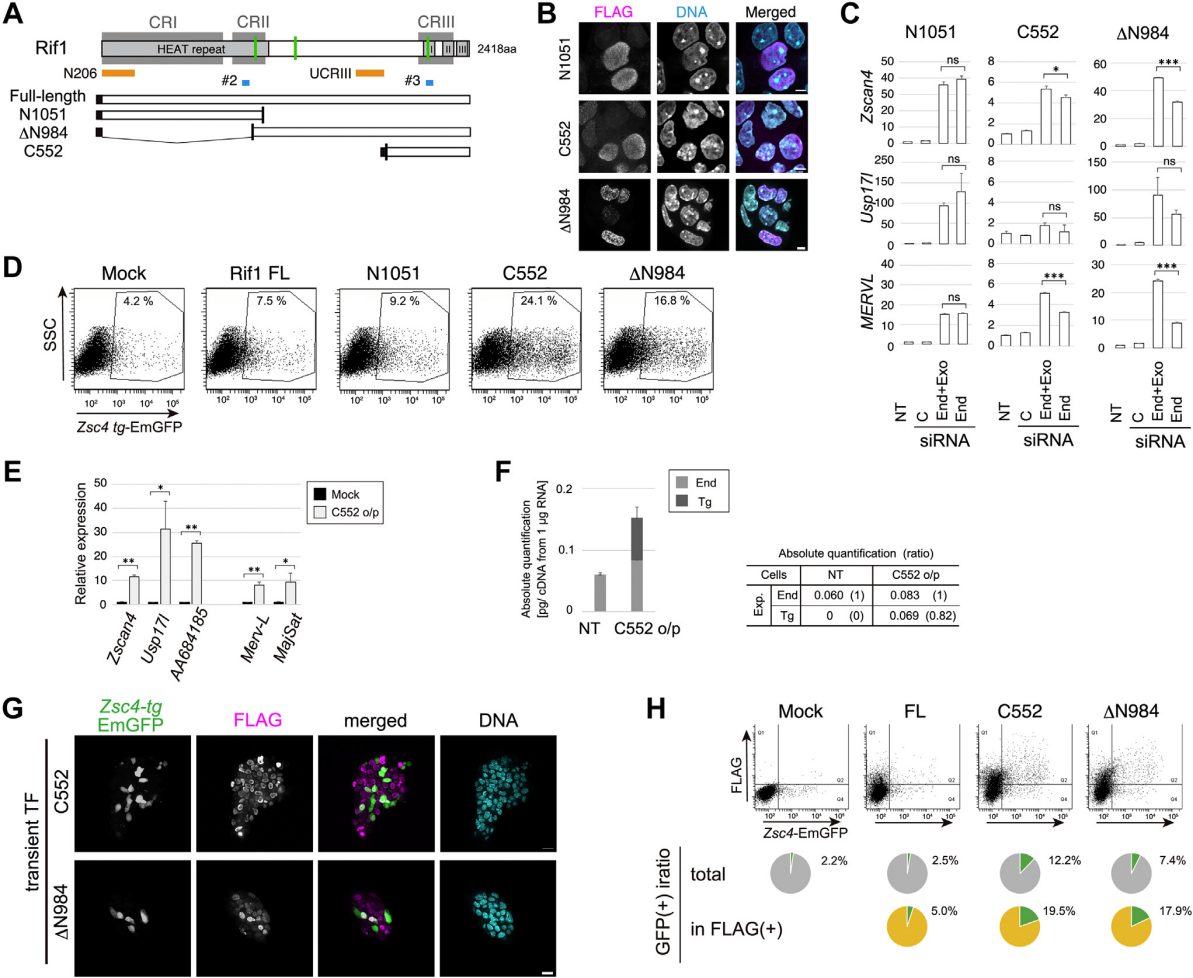
**Ectopic expression of Rif1 deletion mutant C552 and ΔN984 induces Zscan4-positive state.***A*, a schematic of deletion variants used in this experiment. The segments of the polypeptides used as antigens for Rif1 antibodies (N206 for Rif1N and UCRIII for Rif1U, respectively) and those of relevant siRNA-targeting regions (#2 and #3) are indicated. *B*, cellular localization of N1051, C552 and ΔΝ984 polypeptides. E14tg2a cells transiently transfected with FLAG-tagged Rif1 truncated mutants for 48 h were fixed and subjected to immunostaining. DNA was costained with DAPI. Bars, 5 μm. *C*, complementation assay. The stable transformants expressing FLAG-tagged mRif1 N1051, C552 or ΔΝ984 were transfected twice with control siRNA (*C*), siRNAs against both endogenous and exogenous (ectopically expressed) Rif1 (End+Exo; siRNA#2 for N1051, and siRNA#3 for C552 and ΔΝ984), siRNA against only the endogenous transcripts (End; siRNA NC#1) or nontreated (NT). The cells were harvested at 48 h and the relative expression levels of indicated transcripts were measured by qRT-PCR. The means of triplicates are plotted. Bars, SDs. *D*, transient expression of mRif1 deletion mutants and changes in Zscan4(+) populations. *Zsc4-EmGFP* Tg ES cells were transfected twice with indicated mutants or mock treated (Mock) and cells were analyzed by FACS after 51 h. Dot plots of EmGFP are shown. *E*, 2C-specific transcripts are derepressed by Rif1 C552 overexpression. E14tg2a cells transfected with C552 and the relative levels of indicated transcripts were measured by qRT-PCR after 48 h. *F*, the transcripts of endogenous Rif1 (End) and transgene (Tg; transiently transfected FLAG-Rif1 C552) were quantified as described in Experimental procedures. The ratio of Tg expression relative to the endogenous Rif1 expression is shown in the table based on the means of triplicate values. Bars, SDs. *G*, immunostaining patterns of FLAG-Rif1 C552 or ΔΝ984-expressing *Zsc4-EmGFP* Tg mouse ES cell colonies. The cells were fixed at 48 h and immunostained with anti-GFP (Zscan4 reporter) and anti-FLAG (tagged Rif1 proteins). The fluorescent confocal microscopic images are shown. Bar, 20 μm. *H*, representative dot plots of GFP and FLAG signals of *Zsc4-EmGFP* Tg cells transfected with FLGA-tagged Rif1 full-length (FL), C552, ΔΝ984-overexpressed or mock treated, and analyzed by flow cytometry (*top*). The fractions of GFP-positive cells in total (*middle*) or in FLAG-positive populations (*bottom*) are shown in pie charts. Whereas GFP-positive cells are enriched in FLAG-Rif1 C552 and ΔΝ984-positive populations, not all the FLAG-positive cells are GFP-positive. In (*C* and *E*), one out of two sets of technical triplicates data were analyzed by using unpaired two-tailed Student’s *t* test. ∗, *p* < 0.05. ∗∗, *p* < 0.01. ∗∗∗, *p* < 0.001. ns, not significant.

Mammalian Rif1 protein consists of N-terminal HEAT repeat structure, C-terminal conserved domain, and the central long intrinsically disordered polypeptide region (IDR) (Fig. 6*A*). To elucidate the segments required for suppression of 2C gene transcripts, the stable ES cell lines expressing N-terminal 1051 aa, ΔN984 or C-terminal 552 aa of Rif1, all of which localized in nuclei (Fig. 6*B*), were established. When endogenous Rif1 was depleted by siRNAs, none of the three mutants could fully rescue the deregulation of 2C transcript-positive state (Figs. 6*C* and S4*B*). Surprisingly, transient expression of Rif1 C552 or ΔN984 in *Zsc4-EmGFP* Tg cells resulted in significant increase of GFP(+) cells, while full-length and Rif1 N1051 did not affect the GFP(+) population (Fig. 6*D*). Single-cell imaging analysis revealed that signal intensities between ectopic expression of C552 or ΔN984 (FLAG) and GFP were significantly correlated (*p* < 0.01 or 0.05, respectively), suggesting that expression of the C-terminal polypeptides is responsible for the induction of the *Zscan4* transcripts (Fig. S4, *C* and *D*). Furthermore, 2C-specific transcripts including *Zscan4, Usp17l, AA684185, MERVL,* and major satellite, the marks of Zscan4-positive state, were significantly elevated in cells expressing exogenous C552 at the approximately same levels of endogenous Rif1 (Fig. 6, *E* and *F*). Interestingly, not all the populations expressing C552 or ΔN984 became Zscan4-positve (Fig. 6*G*); Only 19.5% or 17.9% of FLAG-positive cells were GFP(+) in C552 or ΔN984-expressing cells, respectively (Fig. 6*H*).

**Figure 7:**
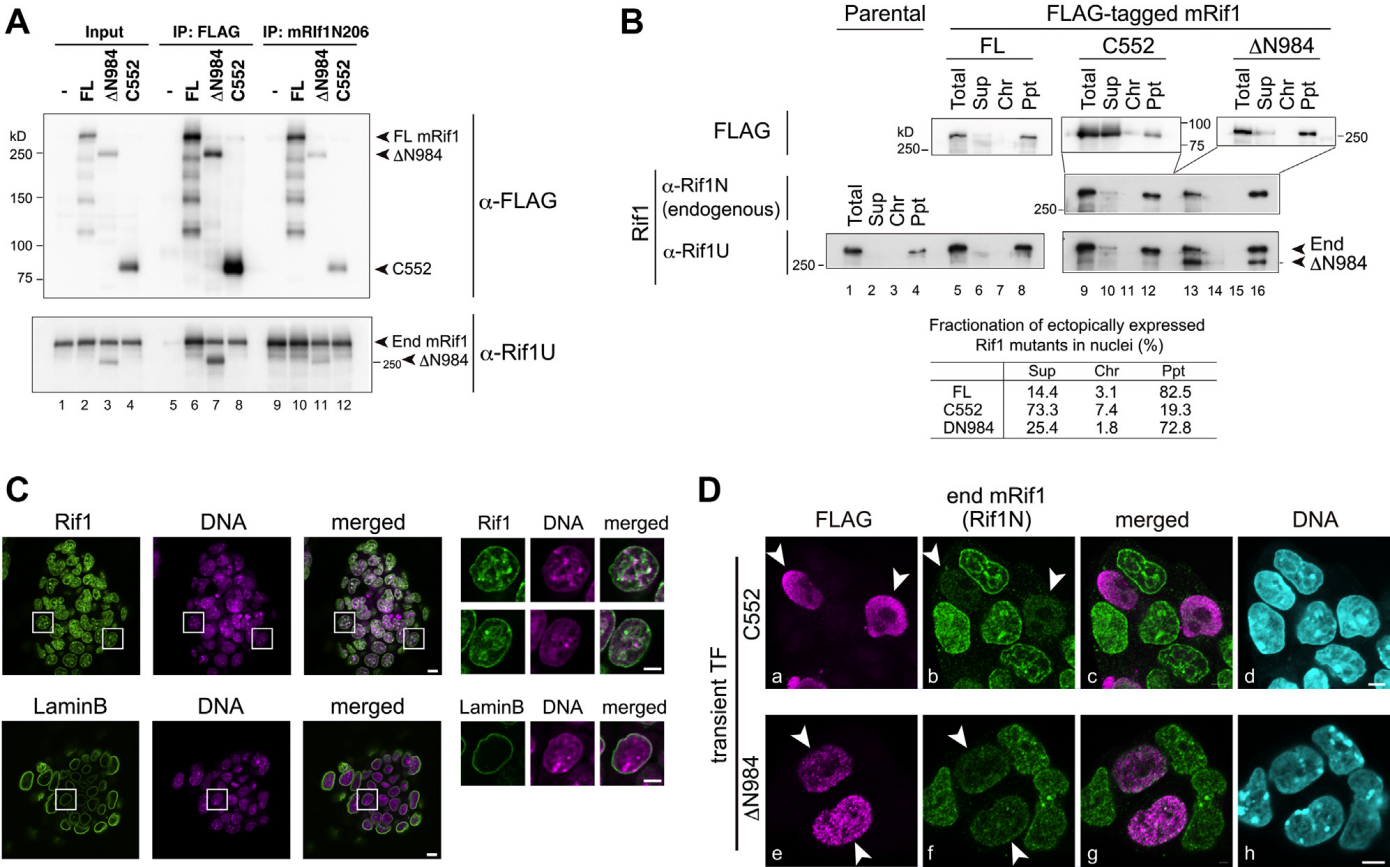
**Rif1 C552 and ΔN984 interact with endogenous Rif1, induce its release from the nuclear insoluble fractions, and compromised signals of the endogenous Rif1 at nuclear periphery and inner nuclear scaffold structures.***A*, immunoprecipitation (IP) of FLAG-tagged exogenous Rif1 and endogenous proteins. IP fractions of FLAG-tagged Rif1 (lanes five–8) or those of endogenous Rif1 (lanes 9–12) were subjected to immunoblot analysis. As input, 4% of each sample was loaded in lanes 1 to 4. *B*, fractionation of endogenous Rif1 and ectopically expressed Rif1 deletion mutants. E14tg2a cells transfected with FLAG-mRif1 full-length (FL), C552 or ΔΝ984 were harvested at 48 h and fractionated as shown in Experimental procedures. Each fraction was resolved by SDS-PAGE and subjected to immunoblot analysis using indicated antibodies. Total, whole cell lysates; Sup, cytoplasmic fractions and nuclear soluble fractions; Chr, chromatin fractions; Ppt, nuclear scaffold-enriched fractions. Quantified fractionation ratios of ectopic Rif1 are shown in the *bottom panel*. *C*, immunofluorescent images of endogenous Rif1. E14tg2a cells were fixed and subjected to immunostaining with anti-Rif1 (Rif1U, *upper panels*) or LaminB (*lower panels*) antibody with DAPI. Enlarged images in *square areas* are shown in *right panel* sets. Bars, 10 μm for original images and 5 μm for enlarged images. *D*, FLAG-tagged Rif1 (FLAG) and endogenous Rif1 (Rif1N) in fluorescent microscopic images. E14tg2a cells transfected with C552 or ΔΝ984 were fixed at 48 h and subjected to immunostaining. DNA was stained with DAPI. Representative images recorded under fluorescent confocal microscopy are shown. *Arrowheads* indicate the cells expressing Rif1 deletion mutants. Bars, 5 μm. Two or more independent experiments were performed for each analysis. Rif1N, anti-Rif1 N206 antibody. Rif1U, anti-Rif1 UCRIII antibody.

To understand the mechanisms underlying derepression of Rif1 by ectopic expression of the N-terminal deletion mutants, we examined its dominant negative effect on the endogenous Rif1. Coimmunoprecipitation assay with anti-FLAG antibody or anti-Rif1 antibody showed that both FLAG-Rif1 C552 and ΔN984 interact with endogenous Rif1 (Fig. 7*A*, lanes 7, 8, 11 and 12). We previously reported that both N-terminal HEAT repeat and C-terminal segments can form homo-oligomers on their own (25). This suggests the possibility that the truncated forms may form hetero-oligomers with the endogenous full-length Rif1, which may be functionally inactive.

Next, to investigate whether overexpression of C552 or ΔN984 would affect the localization of endogenous Rif1, we examined whether it affects the biochemical fractionation and cellular localization of endogenous Rif1. C552, which is exclusively localized to nuclei (Fig. 6*B*), is highly soluble in cellular fractionation analysis (Fig. 7*B*, lane 10; top panel), whereas both endogenous and ectopic full-length Rif1 proteins were distinctively fractionated into nuclear scaffold/matrix fraction like LaminB2 (Fig. 7*B*, lane 4 and 8; Fig. S4*E*). A small amount of endogenous Rif1 was relocated to soluble fraction when C552 was overexpressed (Fig. 7*B*, lane 10; middle and bottom panels). On the other hand, ΔN984 was mostly insoluble with small portions detected in the soluble fraction (Fig. 7*B*, lane 16 and 14 of top panel), and its overexpression did not affect fractionation of endogenous Rif1. We also analyzed the localization using confocal immunofluorescent microscopy. As reported for a human ortholog, the endogenous mouse Rif1 was localized at nuclear membrane and to perinucleolar heterochromatin structures (Fig. 7*C*). In C552-overexpressing cells, the signal intensity of the endogenous Rif1 detected by anti-Rif1 N206 (Rif1N) antibody appear to be compromised, as well as by anti-Rif1 UCRIII (Rif1U) antibody to a lesser extent (Figs. 7*C* and S4*F*; a and b, arrowheads). Similarly, overexpression of ΔN984 reduced the endogenous Rif1 signals (Fig. 7*C* e and f, arrowheads). Since the protein level of endogenous Rif1 was not affected by the ectopic expression of mutant proteins (Fig. 7*A*, bottom panel, lane 3 and 4), the decreased Rif1 signals may reflect structural changes of chromatin and/or nuclear scaffold that block the accessibility of the antibodies to Rif1, or Rif1 protein conformation itself.

### Rif1 localization during mouse early development

**Figure 8:**
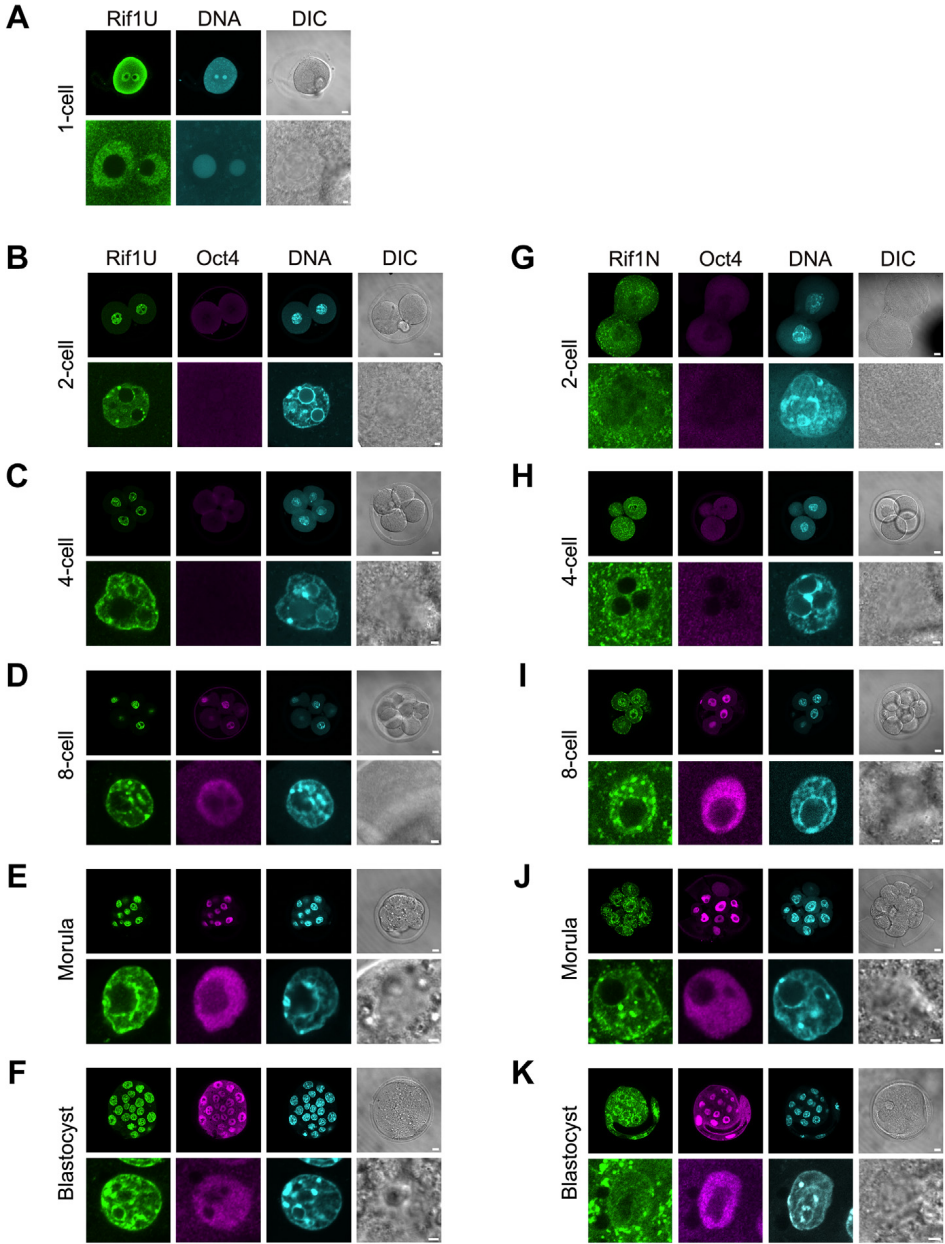
**Cellular localization of Rif1 in preimplantation embryos.** Whole-mount immunostaining of ICR mouse fertilized eggs (*A*) and embryos (*B*–*K*) during preimplantation development using anti-Rif1U (*A*–*F*) or anti-Rif1N (*G*–*K*) antibody, coincubated with anti-Oct4 antibody. DNA was stained with DAPI. In each panel set, an enlarged image acquired by digital zoom at ×3 to ×8 is shown in *lower panels*. Bars, 10 μm (*upper panels*) or 2 μm (*lower panels*). The similar analyses were performed by using C57BL/6J mouse embryos and shown in Figure S5. DIC, differential interference contrast images.

The possible effects of truncated forms of Rif1 on induction of 2C genes prompted us to examine the expression and localization of Rif1 during early developmental stages *in vivo* by indirect immunostaining microscopy using two anti-Rif1 antibodies. In fertilized oocytes of ICR mice, Rif1 accumulated in nuclei and was excluded from nucleolus with diffused week signals in cytoplasm (Fig. 8*A*) as previously reported (5). At the cleavage stage, anti-Rif1 antibody UCRIII (targeting intrinsically disordered/unstructured region (IDR); antibody Rif1U) detected Rif1 clearly and almost exclusively in nuclei, with marked staining at nucleolar periphery and heterochromatin compartments densely stained with DAPI (Fig. 8, *B*–*F*). The signal intensities of Rif1U were mostly unchanged throughout early embryogenesis, whereas Oct4 were markedly decreased at two to four-cell and emerged again after eight-cell stage when zygotic gene became active (39). Unexpectedly, anti-Rif1 antibody Rif1N (targeting the N-terminal 206 aa) showed that the signals were detected in cytoplasm and excluded from nuclei at 2C stage (Fig. 8*G*) and regained its nuclear localization after the four-cell stage (Fig. 8, *H*–*K*). This 2C stage-specific dislocalization of Rif1N signals was also demonstrated in C57BL/6J mouse embryos (Fig. S5*A*), both in early (possibly G1 or S phase) and in late stage (G2 phase) (Fig. S5*B*, b and d, respectively), while Rif1U signals were constantly present in nuclei (Fig. S5*B*, a and c). Inhibition of nuclear export by leptomycin B did not affect the Rif1N signals, suggesting the possibility that Rif1-derived polypeptide containing the N-terminal segment was intrinsically expressed in cytoplasm at 2C stage (Fig. S5*C*).

### Cellular localization of Rif1 and expression of Rif1-derived polypeptides at mouse early developmental stage

**Figure 9:**
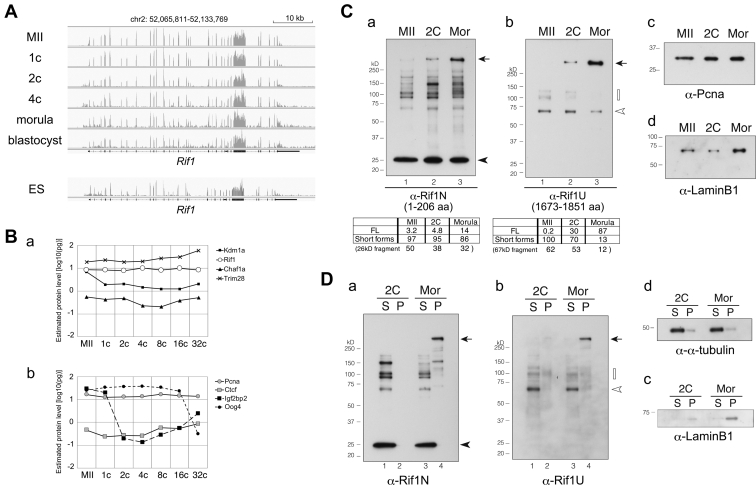
**Rif1 is abundantly present in oocytes and two-cell embryos mostly in truncated and detergent-soluble forms.***A*, RNA-seq data of mouse unfertilized oocytes (MII) or embryos at the different stages of preimplantation development and that of mouse ES cells (40, 81). The mapped reads (the transcript profiles) at the Rif1 gene locus are shown. *B*, the estimated protein levels in oocytes or embryos (41) of Rif1 (*open circles*) and other suppressors of 2-cell specific transcripts, Kdm1a, Chaf1a and Trim28 (filled marks) are shown in (a). Those of chromatin binding proteins with constant expression levels (Pcna and Ctcf; *gray*-filled marks) and stage-specific proteins (Igf2bp2 and Oog4; *dashed lines*) are shown as a comparison (b). *C*, immunoblot analysis of unfertilized oocytes (MII), embryos at two-cell (2C) or at the morula stage (Mor). The whole cell extracts of 120 oocytes or embryos were analyzed by indicated antibodies. As controls, Pcna and LaminB1 are shown. Note that the expression level of LaminB1 in morula increased due to the expansion of cell numbers. In (a and b), the signal intensity ratio (%) of indicated Rif1 protein forms in each lane was shown in the *bottom panels*. Other independent experimental results were shown in Figure S6, *F*–*H*. *D*, cellular fractionation and immunoblot analysis of embryos. The cells were extracted with CSK buffer containing Triton X-100 and separated by centrifugation into the soluble fraction (cytosol and nucleoplasmic proteins; S) and the pellets (P). The proteins derived from 110 (2-cell) or 97 (morula) embryos were analyzed. To validate the fractionation procedure, α-tubulin and LaminB1 were also examined. *Arrows*, full-length Rif1. *Arrowhead*, 25 kD fragments derived from the N-terminal segment. *White arrowhead*, 67 kD fragments lacking the N-terminal segment. *White rectangles*, polypeptides lacking the N-terminal segment and most likely containing the C-terminal segment.

To search for the presence of the alternative forms of Rif1, we first reanalyzed public RNA-sequencing data of mouse oocytes and embryos (40). We have mapped the sequence reads and found that the Rif1 transcripts derived from all exons were present in unfertilized oocytes and in embryos at one-cell to blastocyst stages, constantly and almost evenly (Fig. 9*A*). Next, we retrieved the public data, which were processed from a series of mass spectrometry datasets of mouse oocytes and preimplantation embryos (41). Data was assembled on 8095 protein groups and provided estimation on quantitative protein expression levels. This showed that Rif1 protein was present at relatively high levels and no obvious changes were detected throughout the early developmental stages, from unfertilized oocytes to 32-cell embryos (Fig. 9*B* a). This pattern was similar to Pcna and Ctcf, which were expressed at constant levels (Fig. 9*B* b). Interestingly, the proteins levels of other 2C gene suppressors, including Kdm1a, Chaf1, and Trim28, were also almost constant during these stages (Fig. 9*B* a), whereas Igf2bp2 and Oog4 showed stage-specific changes as previously reported (42) (Fig. 9*B* b). Thus, these transcriptome and proteome data did not provide evidence for the presence of 2C stage-specific short forms of Rif1.

To examine more precisely the expression of Rif1 protein at the 2C stage, we collected mouse oocytes and embryos and investigated the size of Rif1 protein by immunoblot analysis. Two newly developed Rif1 antibodies, Rif1-N206 (Rif1N) and Rif1-UCRIII (Rif1U), react to the full-length Rif1 as a single band in the ES cell extracts (Fig. S6*A*) and detect the ectopically expressed Rif1 fragments with high specificity (Fig. S6, *B*–*D*). These antibodies could detect Rif1 in the extract derived from as few as 100 ES cells (Fig. S6*E*). With these antibodies, Rif1 was detected as multiple short forms and only a small amount of the full-length form was present in unfertilized eggs (estimated to be 3.2% or 0.2% of the total polypeptide detected by Rif1N or Rif1U antibody, respectively; Fig. 9*C*, a and b, lanes 1). In 2C embryos as well, the majority of Rif1 protein was detected as truncated forms. Among them, a 25 kD polypeptide (Fig. 9*C*, a, lane 2, arrowhead) and other forms ranging from 60 to 185 kD constituted 38% and 57%, respectively, of the total polypeptides detected by Rif1N that reacts to N-terminus-derived polypeptides. The antibody Rif1U, which was developed against the central IDR segment upstream of C-terminal conserved domain, detects the 67 kD fragment and other forms (Fig. 9*C*, b, lane 2, white arrowhead) constituting 53% and 17%, respectively, of total Rif1U-reacting proteins. At the morula stage, the full-length Rif1 accounted for 87% of the total polypeptides detected by Rif1U antibody (Fig. 9*C*, b, lane 3). The ratio of the full-length Rif1 to truncated forms appears to continue increasing during the preimplantation stages (from the unfertilized oocytes to one-cell [fertilized egg], 2C, eight-cell, and to the morula stage; Fig. S6, *F*–*H*). The truncation of Rif1 is probably not caused by nonspecific protein degradation during sample preparation, since other proteins, including Pcna and LaminB1, were detected as single full-length forms in the same samples (Fig. 9*C*, c and d).

To characterize Rif1 short forms, the cell extracts were fractionated into the soluble (including cytoplasm and nucleoplasm) and the insoluble (including chromatin and nuclear scaffold) fractions. Most of the short forms in 2C embryo were detected in the soluble fraction, including the major N-terminal 25 kD fragments (Fig. 9*D*, a, lanes 1). In ES cells, the N-terminal Rif1 polypeptide coding N382 aa (43 kD) or N877 aa (95 kD) was localized in cytoplasm (Fig. S6*I*), which coincides with the Rif1N-reacting cytosolic-signals in 2C embryos (Figs. 8 and S5). A major Rif1U-reacting, 67 kD fragment, which is derived from the central portion of the Rif1 protein, was also detected in soluble fractions (Fig. 9*D*, b, lane 1), whereas small amounts of full-length and a minor ∼100 kD forms were bound to insoluble materials (Fig. 9*D*, b, lane 2). At the morula stage, the full-length form was clearly detected in the insoluble chromatin/nuclear scaffold fraction (Fig. 9*D*, b, lane 4). In conclusion, in 2C embryos, most of the truncated forms derived from the N-terminus (less that 95 kD) or from the central IDR segment are not localized in at nuclear scaffolds and thus, are likely to be nonfunctional.

## Discussion

### Rif1 is dispensable for self-renewal and maintenance of undifferentiated state of ES cells, but is required for regulation of Zscan4-positive state

Rif1 is multifunctional protein that plays conserved roles in regulation of replication timing and DSB repair. Rif1 depletion also alters transcription but the effects on transcription profiles are diversely different among species and cell types. The effects of Rif1 on 2C zygotic genes and noncoding transcripts are likely restricted to mouse ES cells and not appreciable in other types of cells ((3, 4, 20); our unpublished result in HCT116 cells). This indicates that transcriptional derepression caused by Rif1 depletion may be influenced by ES-cell-specific chromatin structures and/or factors specifically expressed in ES cells.

Although *Rif1* is one of the major targets of multiple pluripotent transcription factors, we show here that it is dispensable for self-renewal of ES cells and for the maintenance of undifferentiated states, whereas long-term depletion increases partially differentiated cell populations. We also found that Rif1 depletion does not promote differentiation of ES cells to specific lineages under the normal ES culture condition, but it increases the expression of some differentiation markers (*e.g.*, *Sox17, CD34, Fgf5*; Fig. S1*B*). Interestingly, Nanog, one of Rif1-interacting protein (43), is upregulated both at mRNA and protein levels.

Recently, many factors were reported as suppressors of 2C genes or Zscan4-positive state in mouse ES cells. They can be categorized into three groups; (1) Kdm1a (11), Chaf1 (17), Rif1 ((18, 19); this study), miR34a (44), which are 2C gene-specific suppressors, loss of which remarkably promotes cells into Zscan4-positive state. (2) Noncanonical polycomb repressive complex 1.6 components including RYBP (12), Pcgf6 (13), MAX (14); the suppressors of both 2C and meiotic-specific genes. (3) Chromatin regulatory proteins and transcription factors, including G9a, GLP, Trim28/Tif1b/Kap-1, HP1 (45), Tet (46), Dax1/Nr0b1 (16), Zfp42/Rex1 (47), which suppress broad targets, and the depletion of which affects 2C genes in a relatively mild and slow manner. Rif1, belonging to the first group, may share downstream pathway with other members of the same group.

### Epigenetic regulation of suppression of Zscan4-positive state by Rif1 protein: role of DNA methylation

The chromatin structure that underpins the transcriptional bursting of 2C-specific genes in ES cells has not been fully understood. Previous epigenetic analyses of intrinsic Zscan4-positive state of ES cells have indicated modest decrease of DNA methylation in *Tmem92* and *Tdpoz4* loci (33) and global DNA hypomethylation (48). However, DNA demethylation driven by Dnmt knockout or treatment with 5-azacytidine or 5-aza-2′-deoxycytidine does not significantly activate 2C-specific *MERVL*-LTR-driven transcripts. Thus, DNA methylation has been thought to be a consequence but not the cause of the activation of Zscan4/MERVL-positive states (18, 48). In this study, we have shown that DNA of the *Zscan4* gene loci is highly methylated, except for its TSS, and that it is only moderately reduced by Rif1 depletion. In addition, the 3.5 kb upstream region of the *Zscan4d* gene, where *MERVL* is present, also sustains high DNA methylation levels in Rif1 depleted cells, similar to the *Zscan4b/e* loci, which are not associated with *MERVL* in their vicinity. This argues against a role of an endogenous retrovirus in regulation of DNA methylation in the *Zscan4* regions. We conclude that cytosine demethylation does not fully explain derepression of *Zscan4*, although we cannot rule out the effects of cytosine hydroxymethylation. Significant decrease of DNA methylation at *Zscan4d* in Rif1-depleted cells, however, was reported by other group, and this discrepancy may be due to different analytical methods; the use of anti-5-methylcytidine antibody (19) *versus* bisulfite sequencing (this study).

### Epigenetic regulation of suppression of Zscan4-positive state by Rif1 protein: role of histone modification

Histone modification is another important epigenetic regulatory mechanism for activating and silencing gene expression. Based on immunoblot analysis, we have found that Rif1 depletion does not globally change 15 histone modifications, in agreement with previous work reporting no effects on total levels and genome-wide distribution of H3K9me3, H3K27me3, H4K20me3, and H3K4me3 (20). On the other hand, previous works in Rif1-knockdown ES cells indicated a decrease in H3K9me3 but no change in H3K27me3 and H3K4me3 levels (18), or global decrease in H3K9me3 and H3K27me3 and increase in H3K9ac and H3K27ac levels (19). The reason for discrepancy between latter two reports and this work is unclear, but we speculate that immunoblot analyses using histone-enriched fractions enable us to load higher amounts of samples (10–30-fold) and to more accurately detect minor modifications compared with the use of whole cell or nuclear extracts in previous works. Besides, Rif1 knockout in floxed-alleles generally permits more sensitive detection of phenotypic changes than siRNA/shRNA-mediated knockdown system.

Furthermore, our ChIP-qPCR analyses have showed no significant local changes in most of active marks (H3K9K14ac, H3K4me3, H3K4me1, H4K8Ac) and silent marks (H3K27me3 and H4K20me1) of the *Zscan4* gene and neighboring regions, in both Rif1 knockdown and knockout cells with different genetic backgrounds. H3K9me3 of the *Zscan4* gene loci has decreased to some extent (Figs. 3*E* f and S3*B* f), but is still maintained at higher levels than that of *Nanog* (as a control active gene) or the LaminB-associating domain (as a control silent site) in Rif1-depleted cells. ChIP-qPCR analyses of specific genomic loci have advantages in quantitatively mapping histone marks especially for multicopy genes or repeats, when compared with ChIP-seq analysis in which results can be influenced by background signals and/or by multiple parameters in data processing. Importantly, H3K27ac, upregulated in Zscan4-positive ES cells (19, 33), is the only active H3 mark of the *Zscan4* gene and its neighboring regions that changed significantly after loss of Rif1.

### Atypical enhancer structure generated at the *Zscan4* loci after Rif1 depletion

In this study, we have found that Rif1 depletion forms atypical enhancer/promoter structures at the *Zscan4* loci. First, H3K27 acetylation, the only notable histone H3 modification mark in the *Zscan4* region that increases after Rif1 depletion, is not accompanied by another enhancer mark H3K4me1. Generally, H3K4me1 is a primary mark, generating “primed enhancers” decorated only with H3K4me1, which will be converted to “active enhancers” by additional H3K27ac (49, 50). Our results suggest the presence of a novel enhancer chromatin structure marked only by H3K27ac, and this may correlate with rapid and robust derepression of target transcripts. Recent studies have shown that reduction of H3K4me1 at enhancers dose not abolish enhancer activity (51, 52), indicating that H3K4me1 mark is largely dispensable for activating gene expression, although it may have other functions at enhancers (53). Second, unique feature of this *Zscan4* enhancer is its extremely long size. It extends to at least 17 kb, and the genomic sequences of these segments are highly homologous with nine *Zscan4* gene family members, thus H3K27ac modification may span as long as a few hundred kilobases. Recently, “super-enhancers” have been noted as novel chromatin structures for stable and robust expression in ES cells, which widely spread at actively transcribed gene loci in the range of 8.7 kb median size (37). However, the characteristics of “super enhancer” are quite different from those of the *Zscan4* enhancer; the former is densely populated with pluripotent transcription factors (Oct4, Sox2, and Nanog), mediator complexes, and active enhancer marks H3K4me1 and H3K27ac, whereas the *Zscan4* enhancer is associated only with the prominent H3K27ac mark in much longer range of sequences. As an atypical activated domain, pathogenic enhancer bound by BRD4-NUT with hyper H3K27 acetylation signature in NUT midline carcinoma was reported to form “megadomains” raging from 100 kb to 2 Mb (54), although the presence of natural enhancers like this is still unknown. Third, coexistence of the active enhancer mark H3K27ac and semisilent chromatin state (high DNA methylation and moderate H3K9me3 levels). It may be related to its switch-like response between hyperactive and silent states of genes. This “zero-order ultrasensitivity” is a kind of module that is generally described in enzymatic reaction dynamics under saturating conditions such as phosphorylation–dephosphorylation monocycles (55). Similar chromatin modules have been reported in *Drosophila* dorso-ventral patterning enhancers in the poised state, to which both transcriptional activator and repressor are bound, and which are marked with H3K27ac but not with H3K4me1 (56). The maintenance of DNA methylation (semisilent state) in derepressed state coincides with the previous reports on *Zscan4/MERVL*-expression caused by Kdm1a depletion or on spontaneous deregulation events in mouse ES cells (11, 33, 48).

### H3K27ac-independent deregulation of *Zscan4* in Rif1-depleted cells and multiple pathways for suppression of 2C genes

Contrary to local H3K27 acetylation at the *Zscan4* region, global H3K27ac levels did not significantly change although “hyper-H3K27ac” populations were slightly increased. Importantly, hyper-H3K27ac and Zscan4-GFP positive marks are correlated, but the correlation is not 100% (Fig. S3*I*), suggesting that factors other than H3K27ac mark also may affect the Zscan4-positive state. We note two features of Rif1 depletion-induced Zscan4-positive state, not observed in naturally occurring one. (1) The presence of hyper Zscan4-positive populations, which may be caused by strong derepression or long-term persistence of this state. (2) Intact chromocenters (heterochromatin at pericentromeric regions). They colocalize with hyper H3K27ac signals and DAPI-detectable, whereas they are clustered into larger and fewer regions (33) and frequently undetectable with DAPI in naturally occurring Zscan4-positive state. From this result, we speculate that heterochromatin may be only partially derepressed in Rif1-depleted cells unlike intrinsic Zscan4-positive cells. It has been shown that the homeodomain transcription factor, human Dux4, and mouse ortholog Dux are the critical regulators of early zygotic transcripts in ES cells (57, 58, 59), and knockdown of Dux partially reversed reactivation of *MERVL*-reporter caused by Rif1 depletion (19). Since Oct4-negative/chromocenter-negative population, typically induced by Dux overexpression (58), does not appear by Rif1 depletion, we speculate the presence of Dux-independent mechanism for induction of 2C genes. Of note, complete knockout of Rif1 or other 2C gene suppressors (*e.g.*, Kdm1a and Chaf1) cannot provoke 100% transition from the normal to Zscan4/MERVL-positive state (This study; (11, 17)), indicating the presence of multiple additive pathways that lead to suppression of 2C genes, such as response to DNA damage or replication stress (15, 60), or glycolytic activities in ES cells (61).

### Dominant negative effect of C-terminal domain of Rif1 in repression of *Zscan4*

We have also analyzed functional domains of Rif1 using transcription suppression assays in ES cells. Previous study has shown that Rif1 truncation mutant lacking C-terminal domain (ΔC231 aa) largely rescues the Rif1 KO phenotype, but that lacking N-terminal HEAT repeat domain (ΔN1299 aa) does not, leading to the conclusion that N-terminal domain is required for its function (19). In this study, we have shown that Rif1 N1051 (containing the HEAT repeat domain alone) is not sufficient to rescue the Rif1 KD phenotype, indicating that not only the HEAT repeat but also the C-terminal segment is required. Unexpectedly, transient expression of truncated Rif1 containing C-terminal segment (Rif1C) elevates 2C zygotic transcripts. In contrast, the transient expression of the Rif1 N-terminal segment N1051 does not affect *Zscan4* expression. We have recently reported that mouse Rif1 C-terminal 299 aa forms multimers (dimer or dodecamer; (25)), and similarly, fission yeast Rif1 can oligomerize through the C-terminal 91 amino acids (62). We speculate two potential mechanisms for Rif1C-induced *Zscan4* activation. (1) Rif1C exerts dominant-negative effect on endogenous Rif1 by forming nonfunctional hetero-oligomers. (2) The hetero-oligomers may dissociate from nuclear periphery/insoluble nuclear scaffold. The association of Rif1 with nuclear periphery may be crucial for its functions. Notably, 2C transcript-positive state is induced in only a fraction of Rif1-C-overexpressing cell (Fig. 6*H*), as in Rif1-depleted cells.

### Rif1 may not be functional in oocytes and in the two-cell embryos

Using two highly specific Rif1 antibodies, we have found that the N-terminal polypeptide-derived signal is detected only in cytoplasm of the 2C embryos in two different mouse strains and in nuclei after the four-cell stage, whereas the IDR-derived signal is detected in nuclei throughout the cleavage stages of embryos. Unexpectedly, the majority of Rif1 protein appears as truncated short forms in oocytes and 2C embryos, which are likely to be nonfunctional and are dissociated from chromatin. Short Rif1 forms, which are detected by the Rif1-U antibody, could contain the C-terminal segment of Rif1 and exert a dominant negative effect on the remaining full-length Rif1. These Rif1U-reacting short forms, both soluble 67 kD and minor insoluble longer forms, could interact with the residual full-length form (such as Rif1 C552 and ΔN984 in ES cells), may promote dissociation of active Rif1 from nuclear insoluble structures, and/or generate nonfunctional hetero-oligomers, leading to derepression of 2C genes. It should be noted that the presence of the Rif1 short forms is evident in unfertilized oocytes, indicating that the accumulation of short forms alone is not a trigger for zygotic genome activation (ZGA) or 2C gene activation. Nevertheless, the temporal loss of functional nuclear Rif1 at the 2C stage may contribute to formation of permissive chromatin state for zygotic transcription events.

The mechanism for generation of Rif1 short forms is not clear at the moment, but two possibilities can be envisioned. One is alternative splicing and the other is cell-stage-specific protein degradation. Recently, the zygote-specific transcription features were reported; these include genome-wide, promiscuous, or promoter-uncoupled transcription in mouse one-cell zygotes (40) and isoform switching and alternative transcriptional start sites (TSS) in zebrafish zygotes before and after ZGA (63). We could not detect 2C-specific TSSs or exon usage of *Rif1* from public RNA-seq data, although further analysis by long read sequencing or cap analysis of gene expression may clarify the presence or absence of alternative Rif1 transcripts in 2C embryos. In oocytes, maternal proteins/transcripts are required for the events during fertilization, but a large part of them are removed prior to the onset of maternal-to-zygote transition. The two protein degradation systems, autophagy and proteasome-dependent degradation, play important roles in normal development of mouse embryos (64, 65, 66). Rif1, as a suppressor of 2C genes, can be one of the targets of these systems. However, fertilization-dependent protein degradation systems may not function for truncation of Rif1 at the 2C stage, since short forms are present in unfertilized oocytes as well. Thus, other unknown system or distinct proteases (including Calpain or other more specific proteases) may play roles in degrading Rif1.

In summary, we have shown here that the deregulation of 2C genes by Rif1 depletion in ES cells is associated with an atypical enhancer featuring H3K27ac mono-mark and semisilencing chromatin states. In 2C embryos, short forms of Rif1 accumulate, presumably by specific proteolytic degradation or by alternative transcription/splicing. The majority of C-terminally truncated forms are relocating to cytoplasm and truncated forms containing the C-terminal segment can dominantly inhibit the function of the residual full-length Rif1 in nuclei by generating inactive hetero-oligomeric complexes and facilitating its relocation from nuclear or nucleoli periphery. The combined action of cytoplasm relocation and release from nuclear/nucleolar periphery may inactivate Rif1, contributing to expression of 2C-genes. Interestingly, Rif1 localization in ICR blastocysts was like that in the 2C embryos; localization of Rif1 containing Rif1N or Rif1U epitopes is segregated. This suggests occurrence of unknown regulation of gene expression or replication timing during this stage of embryogenesis by inactivating Rif1 through similar mechanisms. We do not detect such phenomenon in ES cells or other cell lines, thus it is oocyte/embryo-specific. Regulation of protein functions by zygote stage-specific alternative transcription and/or by protein truncation and resulting its relocation could be one of the strategies during ZGA and embryo development. Chaf1, 2C gene suppressor, was reported to be dissociated from chromatin only at the early stage of 2C (17). It would be an important future task to clarify the mechanism for generation of truncated forms of Rif1 in a cell-stage-specific manner and to identify other 2C gene regulators that may be regulated in a similar manner.

## Experimental procedures

### Cell culture and transfection

Mouse ES cell line D3 was described previously (67). E14tg2a, a gift from Y. Ruan (Genome Institute of Singapore), less than 36 passage were cultured on gelatin-coated plate without feeder cells and maintained in the ES medium consisting of DMEM supplemented with medium containing 15% fetal bovine serum (Gibco), 4 mM L-glutamine, 0.1 mM 2-mercaptoethanol, 0.1 mM nonessential amino acids, 1 mM sodium pyruvate, and mouse leukemia inhibitory factor (LIF; ESGRO, Millipore; 1 × 10^3^ U/ml). *Zscan4-EmGFP* Tg cells (9) were maintained with the ES medium as above and *Zscan4c-EmGFP* KI (31) were cultured with 15% KOSR (Invitrogen) instead of serum. For transfection, cells dispersed with Accutase (Nacalai Tesque) were used for reverse transfection with plasmid DNA for transient expression or with dsRNA for knockdown, using Lipofectamine2000 (Invitrogen) or FuGENE HD (Roche). In some experiments, cells were dispersed again at 24 h and subjected to second transfection at 2× scale to increase the population of the transfected cells or efficiency of knockdown. *Rif1*^*flox*^^*/f*^^*lox*^ *Cre-ERT2* ES cells were described in “Establishment of inducible Rif1 knockout ES cells” section. Primary mouse embryonic fibroblast cells were isolated from d14.5 C57BL/6J mouse embryos and were maintained in DMEM supplemented with 10% fetal bovine serum, 2 mM L-glutamine, 0.1 mM 2-mercaptoethanol, and 0.1 mM nonessential amino acids. All cell lines in this study were regularly tested for the absence of *mycoplasma* contamination.

### Animals

ICR (CD-1) and C57BL/6J mice were purchased from Charles River Laboratories Japan and CLEA Japan, respectively. C57BL/6J *Rif1* ^*flox/flox*^ mouse embryos were provided by the Rockefeller University Comparative Bioscience Center. All animals were treated in conformity with the Guidelines for Proper Conduct of Animal Experiments (Science Council of Japan) and the ARRIVE guidelines, and all studies using animal experiments were approved by the Animal Care and Use Committees of Tokyo Metropolitan Institute of Medical Science (permission Nos. 20-030 and 21-022).

### DNA constructions

Three fragments of mouse *Rif1* cDNA were isolated from D3 cells and cloned into pBluescript KS II (−), and the sequence was validated from three independent clones (GenBank: JX255737). Resulting sequence is highly homologous to AY428571 derived from ES cells established from 129/Sv strain (5). The backbone expression vector pPyCAG-2×FLAG-IRES-puro (pCAGIP-FLAG) was generated by inserting 2×FLAG adaptor (amplified by PCR with Flag-F_MfeI and Flag-R_RI primers in Table S1 using cDNA3.1(-)-FLAG×2 (68) as a template) at EcoRI site in the original vector (69), followed by a removal of SV40 Ori at two BamHI sites and self-ligation. Rif1 full-length expression vector was generated by two-step cloning; First, *Rif1* cDNA fragment 5596 to 7257 bp, amplified by Phusion DNA polymerase (Finnzymes) with C552-F primer (containing substitution of G5598A to generate BamHI site as a silent mutation) and C552-R primer (containing XhoI and NotI site) using pBluescript-mRif1 C552aa as a template, was cloned to backbone vector at EcoRI site. Second, *Rif1* cDNA 1 to 5595 bp was clone to this vector at EcoRI site using In-Fusion (Clontech). Rif1 C552 expression vector was derived from the products of the first step by digestion with BamHI, blunting by T4 DNA polymerase and self-ligation to make it in frame. Rif1 N1051 expression vector was generated from full-length construct by deletion of cDNA 3153 to 5497 bp (at EcoNI site 3152 and 5497 cut by EcoNI star activity) resulting in the coding N-terminal 1051 aa with additional two amino acids at the C-terminus. Rif1 ΔN984 was also derived from the full-length constructs by deletion of 1 to 2951 bp and self-ligation at EcoRI sites. For Rif1 N877 expression vector, *Rif1* cDNA fragment 1 to 2631 bp was amplified by PCR with mRif1-N(RI)frame3 and mRif1_2626R_SpeI primers and cloned into pME18S. Then the insert DNA was isolated by digestion with EcoRI and NotI and cloned into pCAGIP-FLAG. Rif1 N382 expression vector was derived from pCAGIP-FLAG-N877 by digestion with XhoI and NotI, blunting by Mung Bean nuclease and self-ligation. Rif1 N1307 was generated from N1051 expression vector by digestion with EcoNI and NotI followed by insertion of Rif1 cDNA 3139 to 3921 bp amplified with mRif1_EcoNI_F3139 and mRif1_R3921stop primers by using In-Fusion. Rif1 ΔN782 was generated from ΔN984 vector, by insertion of *Rif1* cDNA 2347 to 2967 bp amplified with mRif1_F2347_Flag_IF and mRif1_R2967_IF primers by In-Fusion. Since plasmid DNA containing *Rif1* cDNA full-length and the N-terminal coding regions were highly unstable in bacterial cells for unknown reasons, transformation and culture have been carried out at 25 to 30 °C in XL10-Gold (Stratagene) and expanded at least three clones per constructs. The sequences were validated for every preparation before the use in the experiments.

For bacterial expression of GST-fused mouse Rif1 N206 aa protein, N-terminal Rif1 coding region (AY428571) was amplified by PCR with mRif1_N_BamHI and mRif1_R1137_XhoI primers (shown in Table S1) using pBluescript II SK(+)-full length Rif1 cDNA derived from R1 mouse ES cells (GenBank AY428571; a gift from I. Adams) as a template, and cloned into pGEX-4T1 (Pharmacia) at BamHI and XhoI sites, then 619 to 1137 bp of CDS was removed by digestion with XbaI and NotI, followed by blunting by Mung Bean Nuclease (Takara) and self-ligation. For GST-Rif1 1673 to 1851 aa protein expression vector, cDNA fragment was amplified with mRif1_F5017_BamHI and mRif1_R5553_XhoI primers using the same template, and cloned into pGEX-4T1 at BamHI and XhoI sites.

pZDonor-mRosa26-ZNFTSmut-SA-CreERT2-IRES-Puro vector was constructed as follows; First, three point mutations in zinc finger nuclease (ZFN) recognition sequences was introduced to original pZDonor-mRosa26 vector (Sigma-Aldrich) by PCR mutagenesis (at A4538 to G, 4541C to G and 4545T to A) to prevent cleavage of integrated donor alleles. The PCR fragment amplified by using F primer with intact sequence and R primer containing mutations was ligated to at NotI and XmaI sites, resulting pZDonor-mRosa26-ZFN-TSmut. Second, SA-*CreERT2*-HSV thymidine kinase polyA digested from pMB80 vector (Addgene) was cloned into pBluescript II KS(−) at XbaI and EcoRI sites, followed by deletion of polyA at MluI and EcoRI sites and self-ligation. Third, IRES-*pac*-bGH polyA digested from pPyCAGIP vector at EcoRI and BamHI (blunted by T4 DNA polymerase) was ligated at EcoRI and blunted SalI sites of second construct, resulting pBluescript II KS(−)-SA-*CreERT2*-IRES-*pac*-bGH polyA. Finally, SA-*CreERT2*-IRES-*pac*-bGH polyA in third construct was cloned into first construct pZDonor-Rosa26-ZFNTSmut by In-Fusion. All primers used for constructions are listed in Table S1.

Three conserved regions (CRI, II, and III) and HEAT repeat regions of Rif1 protein were assigned from previously reported human Rif1 sequence (21, 24). Nuclear localization signals were predicted by MotifScan by EXPASY (https://www.expasy.org/). IDR was predicted by PredictProtein (70).

### Antibodies

Recombinant proteins, mouse Rif1 N206 aa or Rif1 UCRIII (the upstream segments of C-terminal conserved region III (24); 1673–1851 aa), were expressed in ER2566 cells (New England Biolabs) transformed with pGEX-4T1-N206 or pGEX-4T1-UCRIII by induction with 0.1 mM IPTG at 30 °C for 3 h. The proteins were solubilized by sonication of cells using Sonifier 250D (Branson) on ice in PBS supplemented with 1% Triton X-100, protease inhibitors (1 μg/ml pepstatin A, 1.5 μg/ml aprotinin, 1 μg/ml leupeptin (Sigma-Aldrich), 0.4 mM Pefabloc SC (Roche)), and 5 mM DTT. After removal of insoluble materials by centrifugation at 60,000*g* for 20 min at 4 °C, GST-fused proteins were purified using glutathione Sepharose 4B (GE Healthcare). For generation of polyclonal antibodies, 0.3 mg of purified proteins was used to immunize a rabbit each time for total five times, and two animals for each antibody were used (Bio Regenerations). The antibodies were affinity-purified by AKTA Explorer 10S (GE Healthcare) using recombinant antigen protein-conjugated HiTrap NHS-activated HP columns following manufacturer’s instructions and validated the specificities. Other antibodies used in this study are listed in Table S2.

### Differentiation of ES cells

Tetracyclin-inducible Oct4-knockout cell line ZHBTc4 and Oct4-overexpresssing ZHTc6, gifts from Hitoshi Niwa, were cultured as in previous report (28) in the presence of 6 μg/ml Zeocin for the maintenance. To deplete Oct4, ZHBTc4 cells were incubated with doxycycline (Dox; 1 μg/ml) for 7 days. To overexpress Oct4, ZHTc6 cells, precultured in the presence of Dox, were transferred to medium without Dox. Differentiation of ES cells by embryoid body formation was conducted as previously reported (71) with modifications; 4 × 10^2^ E14tg2a cells were cultured in hanging drops on bacterial plate cover for 4 days and transferred to bacterial plates with ES medium in the absence of LIF. On day 6, cells were dispersed with trypsin and cultured for a day, and then cells were cultured for additional 5 days in medium containing 1 mM retinoic acid. Cells were harvested at indicated timing in Figure 1*C*.

### qRT-PCR and complementation analysis

Total RNA extracted from ES cells with TRIZOL (Invitrogen) was purified by 2-propanol precipitation and quantified by NanoDrop (Thermo Fisher). After DNase treatment of 1 μg or 0.5 μg of total RNA, cDNA was synthesized from RNA with PrimerScript RT reagent Kit (Takara) or ReverTra Ace qPCR RT Master Mix (Toyobo). One to two microliters of tenfold diluted cDNA was subjected to real-time PCR using SYBR Premix Ex Taq (Takara) on LightCycler 480 (Roche). PCR cycle condition is; 95 °C for 1 min, followed by 45 cycles of 95 °C for 5 s and 68 °C for 30 s for most of primer sets, or 45 cycles of 95 °C for 10 s, 57 °C for 20 s and 72 °C for 16 s, for others. The relative quantification values were normalized by *Gapdh*. For absolute quantification, pPyCAG-FLAG-IP-full-length Rif1 was quantified by Qubit (Invitrogen) and used as a PCR template for a standard curve, and the amounts of total *Rif1* (using the primers targeting both endogenous and transgene) or those of transgene (using the primer for 3′ UTR of transgene) were calculated for picograms of cDNAs derived from 1 μg total RNA (Fig. 6*F*).

For complementation assay, E14tg2a stable transformant cells expressing full-length Rif1, N1051, C552, or ΔN984 were generated by transfection of plasmid vectors and selected by 2 μg/ml puromycin. The clones with levels of ectopic expression of Rif1 transgenes similar to those of the endogenous gene were used for analysis. The effects on derepression of 2C transcripts between cells depleted of endogenous Rif1 alone (using siRNA targeting 3′-UTR, siRNA NC#1) and those of both endogenous and transgene (using siRNA #2 or #3) were examined. For all qRT-PCR analysis, a minimum of three independent experiments were done, in technical triplicate. All primers used for qRT-PCR are listed in Table S1.

### Chromatin immunoprecipitation (ChIP) and ChIP-qPCR

E14gt2a cells were transfected with control or Rif1#2 siRNA twice and cultured for 72 h, or *Rif1*^*f/f*^ *Cre-ERT2* mouse ES cells treated with or without 4-OHT were cultured for 60 h. Fixed chromatin was prepared as previously reported (72) with modifications. Chromatin of Rif1-depleted or untreated cells was cross-linked *in situ* with 1% formaldehyde in PBS for 10 min at RT under rotation, then quenched with 135 mM glycine for 10 min. Cells were rinsed twice with cold PBS, harvested with a silicone rubber-attached scraper, lysed twice with FA-lysis buffer (50 mM HEPES-KOH pH7.5, 150 mM NaCl, 0.1% SDS, 0.1% sodium deoxycholate, 1% Triton X-100, 1 mM DTT, 1 mM Na_3_VO_4_,) containing protease inhibitors (1 μg/ml pepstatin A, 1.5 μg/ml aprotinin, 1 μg/ml leupeptin (Sigma-Aldrich), 0.4 mM Pefabloc SC (Roche)) at 4 °C for 15 min, and centrifuged at 440*g* for 10 min. The pellets were lysed with Nuclear lysis buffer (FA-lysis buffer containing 1% SDS instead of 0.1% supplemented with protease inhibitors) at 4 °C for 15 min, and nuclei was centrifuged at 1750*g* (for Rif1 conditional knockout cells) or 440*g* (for E14tg2a cells) for 10 min. Chromatin pellets were washed and resuspended with FA-lysis buffer (at the density of nuclei derived from 1 to 2 × 10^7^ cells/ml) in 15 ml polypropylene tubes (Corning), and sequentially sonicated by Sonifier 250D (Branson) on ice at 20% with 50% frequency (1 s on and 1 s off) for 1 min, 2 to 5 times with 2 min intervals, followed by Covaris S-220 (Covaris) in 1 ml glass tubes at power 150W to 175W, Duty Factor 20, for 12 to 15 min at 4 °C. The size of sheared chromatin was assessed by agarose gel electrophoresis, ranging from 100 to 400 bp with average size at 200 bp. After centrifugation at 39,000*g* for 10 min and a preclear by beads for 6 h at 4 °C, the sonicates corresponding to 1 × 10^6^ cells were diluted to 0.5 ml with FA-lysis buffer containing 0.5 mM DTT and protease inhibitors, and incubated with 1 μg of histone antibodies bound to 20 μl of Dynabeads Protein G (Life Technologies) for 12 to 16 h. The beads after ChIP were washed at 4 °C for 5 min with 1 ml of buffers as follows: with FA-lysis buffer without protease inhibitors (three times), FA-lysis buffer containing 350 mM NaCl, ChIP wash buffer (10 mM Tris-HCl pH 7.5, 0.25 M LiCl, 1 mM EDTA, 0.5% Nonidet P-40, 0.5% sodium deoxycholate), and TE (once for each step). DNA was eluted with 0.3 ml of ChIP elution buffer (50 mM Tris-HCl pH 7.5 and 10 mM EDTA, 1% SDS) at 37 °C for 30 min on rotary mixer (TAITEC) with constant tapping. The eluates were incubated at 65 °C for 6 h for de-cross-linking, treated with RNaseA (50 μg/ml) at 37 °C for 1 h and treated with proteinase K (0.5 mg/ml) at 50 °C for 2 h. DNA was extracted with phenol/chloroform/isoamyl alcohol, and the organic phase was re-extracted with TE containing 0.2 M NaCl. The combined aqueous phase was added with 1× vol of 2-propanol, 0.3 M sodium acetate and glycogen, and centrifuged at 17,000*g* for 30 min. DNA was repurified by MinElute (QIAGEN) and eluted with 50 to 80 μl of EB (Qiagen). One microliter of ChIP DNA (approximate 5 ng) was subjected to ChIP-qPCR using SYBR Premix Ex Taq (Takara) on LightCycler 480 (Roche). PCR condition for most of primer sets was: 95 °C, 30 s, followed by 45 cycles of 95 °C, 7 s and 62 °C, 30 s; for Zscan4-ChIP4, 45 cycles of 95 °C, 7 s and 62 °C, 40 s; for T-ChIP, 45 cycles of 95 °C, 7 s, 57 °C, 15 s and 72 °C, 10 s. Input samples were de-cross-linked and purified as above, and the ChIP efficiency (ratio of ChIP DNA to input DNA) was calculated. All experiments were done at least two times, with technical triplicates. All primers used for ChIP-qPCR are listed in Table S1.

### DNA methylation analyses

DNA methylation analyses by HpaII-digestion/PCR were previously reported (73). Briefly, genomic DNA of Rif1-siRNA treated or untreated ES cells was prepared by a standard protocol with RNaseA and Proteinase K treatment followed by phenol/chloroform extraction and ethanol precipitation. Purified genomic DNA (200 ng) was digested with HpaII or untreated, and amplified by PCR using specific primers under semiquantitative conditions (29 cycles for *Fgf5*; 30 cycles for *Zscan4e*, *Bmp4*, and *T*). The products were analyzed by gel electrophoresis using 2% (*Bmp4*) or 4% (*Zscan4e*, *Fgf5*, and *T*) agarose in TBE buffer. As a control, methylation-insensitive isoschizomer MspI was used for digestion. Two independent experiments were performed.

DNA methylation was analyzed also by targeted amplicon bisulfite sequencing by previous protocol (74). Denatured genomic DNA (2 μg) was treated with sodium bisulfite solution at 50 °C for 6 h and purified by MinElute cleanup kit (Qiagen). The treated DNA was amplified by using specific primer sets listed in Table S1 and cloned into pGEM-T vector (Promega). The sequences were analyzed using 3500XL Genetic Analyzer (Applied Biosystems). To accurately quantify methylation ratio in BS1, BS2, and BS3 regions, clones were classified into each *Zscan4* gene family members and compared with corresponding genomic sequences, in order to distinguish between converted and intact TpGs, which were derived from polymorphism among gene family members. For BS2 region, 29 clones were sequenced, and only those derived from the *Zscan4b*, *e*, and *d* loci, which contain CpG sites, were shown. Since BS4 region in the *Zscan4* exon 4 is well conserved, clones were not classified but compared with the *Zscan4c* reference sequence. Two independent experiments were performed. All primers used for DNA methylation analysis are listed in Table S1.

### Establishment of inducible Rif1 knockout ES cells

ES cells were derived from blastocysts of C57BL/6J *Rif1*^*f/f*^ mice (75) as reported previously (76). Three clones among 17 lines were subjected to adaptation from ES derivation medium consisting of DMEM-F12/Neurobasal/N-2/B27 to KOSR ES medium as previously reported except for the presence of 0.8 μM PD0325901 and 2.4 μM CHIR99021. Two adapted lines #5 and #12 were transfected with pZDonor-mRosa26-ZNFTSmut-SA-CreERT2-IRES-Puro and ZFN mRNA-mRosa26 (Sigma-Aldrich) using Lipofectamine2000 (Invitrogen) and stable clones of tamoxifen-inducible Rif1 knockout cell line #5-17 and #12-9 were isolated. To deplete Rif1, the cells were incubated with 0.2 μM 4-hydroxytamoxifen (4-OHT) for 48 to 72 h.

### Flow cytometry analysis

For live cell analysis, dispersed ES cells were washed with PBS and suspended with FACS buffer (PBS/2% fetal bovine serum/0.02% NaN_3_), and the EmGFP signals were analyzed by FACSCantoII or LSRFortessa X-20 (Beckton Dickinson) at FITC channel. For immunostaining analysis, cells were fixed with 4% paraformaldehyde for 15 min, permeabilized with FACS buffer containing 0.5% Tween 20 were incubated with anti-H3K27ac or anti-FLAG M2, and with anti-GFP antibodies at 4 °C for overnight. The cells were washed with FACS buffer containing 0.1% Tween 20 three times followed by incubation with secondary antibodies, PE-conjugated anti-mouse IgG (BD Pharmingen), and Alexa Fluor 488-conjugated anti-rat IgG (Invitrogen), for 30 min. Washed samples were analyzed by LSRFortessa X-20 (Beckton Dickinson). Data was analyzed by using BD FACSDiva software (Becton Dickinson). Each analysis was done in two to five independent experiments with biological triplicates for each set of experiment.

### Cellular fractionation

ES cells were fractionated as previously described (77) with minor modification. Briefly, 3.6 × 10^6^ cells were dispersed with Accutase, washed with PBS, suspended with 200 μl of CSK buffer (20 mM HEPES-KOH, pH 7.6, 100 mM NaCl, 300 mM sucrose, 1 mM MgCl_2_, 1 mM EGTA, 300 mM sucrose, 20 mM NaF, 1 mM DTT, 1 mM Na_3_VO_4_, and protease inhibitors) containing 0.1% Triton X-100, lysed on ice for 5 min, and centrifuged at 800*g* for 3 min at 4 °C. The pellets were washed again with 100 μl of CSK buffer without detergent, and the first and second supernatants were combined and stored as cytosol and nuclear soluble materials. The precipitates were suspended with hypotonic buffer (3.75 mM Tris-HCl, pH 8.0, 10 mM potassium acetate, 0.5 mM EDTA, 2 mM MgCl_2_, and 1 mM Na_3_VO_4_) supplemented with protease inhibitors and 100 U/ml of Benzonase (Novagen), and incubated on ice for 1 h. After addition of 10 mM EDTA, the samples were separated into the supernatants (chromatin-enriched fractions) and the precipitates (nuclear scaffold-enriched fractions). Five percent of each fraction was analyzed. Two independent experiments were performed for ES cell fractionation.

For cellular fractionation of embryos, 220 2C embryos or 193 morula embryos were collected in 6 to 8 μl of KSOM without BSA and freshly frozen. The samples were thawed by addition of 1× volume of 2× CSK+ buffer (40 mM Hepes-KOH, pH 7.6, 100 mM NaCl, 600 mM sucrose, 1.8 mM MgCl2, 2 mM EGTA, 40 mM NaF, and 0.2% Triton X-100 supplemented with protease inhibitor cOmplete (Roche)), and the total volume was adjusted to 30 μl with 1× CSK+ buffer (20 mM Hepes-KOH, pH 7.6, 100 mM NaCl, 300 mM sucrose, 1 mM MgCl_2_, 1 mM EGTA, 20 mM NaF, and 0.1% Triton X-100 with cOmplete). The samples were transferred to siliconized polypropylene 0.2 ml tubes, suspended with low retention pipet tips, and incubated on ice for 5 min. The samples were centrifuged at 800*g* for 5 min, at 4 °C in a swing rotor, and collected the 25 μl of supernatants. To the pellets and remaining supernatants, 15 μl of 1× CSK+ buffer was added and suspended again by pipetting. After centrifugation, the supernatants were combined and added with 0.25× volume of 5× sample buffer. To the precipitation fraction, ×4 volume of 1.25× sample buffer was added. The samples were boiled, and each half of them was loaded onto an acrylamide gel, and two sample sets were subjected to immunoblot analysis.

### Histone modification analysis

Histone-enriched fractions were prepared from *Rif1*^*f/f*^ *CreERT2* cells cultured with or without tamoxifen for 48 h by acid-extraction methods (78) with modification. Briefly, 1.2 × 10^7^ cells were washed with 0.5 ml of nuclear isolation buffer (NIBi: 15 mM Tris-HCl, 60 mM KCl, 15 mM NaCl, 5 mM MgCl_2_, 1 mM CaCl_2_, and 250 mM sucrose, pH 7.5, supplemented with 1 mM Na_3_VO_4_, 1 μg/ml pepstatin A, 1.5 μg/ml aprotinin, 1 μg/ml leupeptin (Sigma-Aldrich), 0.4 mM Pefabloc SC (Roche), and 10 mM sodium butyrate), centrifuged at 700*g*, and homogenized with NIBi plus 0.2% NP-40. Cells were incubated on ice for 10 min and centrifuged at 1000*g*. After two-time wash of nuclei with NIBi without detergent, the pellets were suspended with 0.2 ml of chilled 0.2 M H_2_SO_4_ and rotated at 4 °C for 4 h. The supernatants were collected by centrifugation at 3400*g* twice and 100 μl of chilled 100% TCA was added and incubated on ice for overnight. The TCA precipitates were washed with cold acetone +0.1% HCl and then with acetone. The dried pellets were suspended in water, and cleared supernatants were quantified by Bradford assay. In total, 3 to 0.1 μg of histone-enriched extracts, for modified histones or pan H3/H4, respectively, were boiled in SDS-PAGE sample buffer and loaded on 15% acrylamide gels, and resolved by SDS-PAGE. The blots were incubated with blocking solution (5% BSA in TBS-T) for 1 h, then incubated with primary antibodies diluted in blocking solution for 2 h at RT. Chemiluminescent detection was done as described elsewhere. For control experiments, E14tg2a cells were treated with trichostatin A (5 μM, 16 h; Sigma-Aldrich), 5-aza-deoxycytidine (5 μM, 48 h; Tokyo Chemical Industry). At the harvest, cells were washed using PBS with or without 10 mM sodium butylate. The histone-enriched fractions were prepared by acid extraction methods and analyzed by immunoblot as above. The experiments were done 2 to 4 times, depending on target marks.

### Immunoblot and immunoprecipitation analysis

For preparation of whole cell extracts, the cells were lysed with lysis buffer A (20 mM HEPES-NaOH, pH 7.4, 1 mM EDTA, 0.1 mM EGTA, 1 mM MgCl_2_, 150 mM NaCl, 1 mM Na_3_VO_4_, 20 mM NaF, 5% glycerol, 1% Nonidet P-40, 1 μg/ml pepstatin A, 1.5 μg/ml aprotinin, 1 μg/ml leupeptin, and 0.4 mM Pefabloc SC) containing 0.3 M NaCl (buffer A/0.3 M) (77) and quantified by Bradford protein assay (Bio-Rad). The amounts of 10 μg (unless otherwise noted) per each sample were resolved by SDS-PAGE and subjected to immunoblot analysis. For immunoprecipitation, transfected cells were lysed with buffer A/0.3 M for 30 min at 4 °C and centrifuged at 20,400*g* for 10 min. The supernatants were diluted to 150 mM NaCl and precleared by incubation with Sepharose 4B (GE Healthcare) by rotation for 30 min. The lysates containing 0.5 mg proteins were incubated with 30 μl of anti-FLAG M2 agarose (Sigma-Aldrich) or 1 μg of anti-Rif1N antibodies followed by precipitation with 30 μl of protein A/G (2:1) Sepharose (GE Healthcare). The beads were washed with buffer A containing 150 mM NaCl for 4 times and the boiled samples were divided into 2 to load on 7% polyacrylamide gels. For immunoblot analysis, samples were transferred to Immobilon-P (Merck) membranes, and blots were incubated with blocking solution (5% nonfat milk in TBS-T) for 30 min, then incubated with primary antibodies for 2 h at RT. After incubation with horseradish peroxidase (HRP)-conjugated secondary antibodies (Jackson ImmunoResearch) for 45 min, chemiluminescent signals were developed by using Lumi-Light PLUS (Roche) and detected by LAS 3000 (Fuji film) or FUSION FX7.EDGE (Vilber Lourmat). The signal intensity of bands was quantified by Multi Gauge software version 3.0 (Fuji Film). All experiments were done at least two times.

One hundred and twenty mouse unfertilized eggs, 2C or morula embryos were collected by counting under microscopy and snap-frozen in liquid nitrogen. The samples were directly suspended in SDS-PAGE sample buffer supplemented with protease inhibitors described above and heat-denatured at 95 °C, 5 min. After agitation by microtube mixer for 5 min to shear chromatin DNA, the whole extracts were loaded onto 5 to 20% acrylamide gradient gel e-PAGEL (ATTO) and resolved. The proteins were transferred to membranes by tank blotting system (BIO CRAFT). The membranes were incubated with 5% skim milk in TBS-T for 1 h and incubated with anti-Rif1N antibody for 4 °C overnight, then incubated with secondary antibody (HRP-conjugated goat whole IgG, anti-rabbit or anti-mouse IgG (H + L), Jackson ImmunoResearch) in 1% Blocking reagent (Roche) in TBS-T for 45 min. The signals were detected as above and exposed to X-ray films. After detection, antibodies were removed by shaking in stripping buffer (62.5 mM Tris-HCl, pH 6.8, 2% SDS, and 46 mM 2-mercaptoethanol) at 50 °C for 6 min, and the membranes were used for reblotting with different antibodies. In experiments in Figure S6, *F*–*H*, 150 cells of fertilized eggs (6 hpf), 2C or eight-cell embryos were used and processed as above. Two independent experiments were done for oocyte/embryo immunoblot analysis. The size of Rif1 truncated forms was calculated by migration distances to be fit to logarithmic curve models.

### Immunofluorescent staining

Preparation of cells for immunofluorescence staining was performed as previously described with modifications (77). Briefly, untreated or transfected ES cells were cultured on embryonic fibroblast feeder cells for 24 to 48 h before analysis and fixed with 4% paraformaldehyde in PBS for 15 min. After permeabilization with PBS containing 0.2% Triton X-100 for 5 min and blocking with 3% BSA, cells were incubated with primary antibodies diluted with AB buffer for 1 h at 37 °C or at 4 °C overnight. After three times washes with PBS, cells were incubated with Alexa Fluor dye-conjugated or Cy3-conjugated secondary antibodies (listed in Table S2) and 0.5 μg/ml of DAPI (Sigma-Aldrich) for 45 min at 37 °C. The coverslips were then washed with PBS three times and mounted with PBS containing 90% glycerol and 2.5% 1,4-diazabicyclo(2.2.2)octane (DABCO; Sigma-Aldrich). For all immunofluorescent staining analyses, two or more independent experiments were performed.

### Fluorescence, phase-contrast, and alkali phosphatase staining microscopy

Fluorescent images were acquired with LSM780 confocal laser scanning microscope (Carl Zeiss) and objective lens (PlanApo20×/0.8, PlanApo40×/1.3 or PlanApo63×/1.4). Alexa Fluor 488 was detected using the 488 nm argon laser and a 495 to 551 nm filter; Cy3 was detected using the 561 nm DPSS laser and a 569 to 630 nm filter; DAPI was detected using a 405 nm blue diode laser and a 410 to 483 nm filter. Conventional fluorescent images were acquired with microscope BX-61, UPlanSApo 40×/0.95 lens (Olympus), and ORCA R2 CCD camera (Hamamatsu Photonics). For phase-contrast images, data were captured by microscope CKX41 (Olympus) equipped with COOLPIX P6000 (Nikon). For alkali phosphatase staining, samples were processed by alkali phosphatase kit (Sigma-Aldrich) according to provider’s instruction except for using 4% paraformaldehyde instead of citrate buffered acetone for cell fixation. Stained cell images were recorded using IX-71 inverted microscope equipped with digital camera DP72 (Olympus). The levels of images were adjusted by Photoshop in some data series. Brightness and contrast were not adjusted. Two independent experiments were performed for alkali phosphatase staining.

### Gene expression analysis by microarray

E14tg2a cells were transfected with control siRNA or mRif1#2 plus NC#1 and harvested at 48 or 72 h. Total RNA was purified from transfected or untreated cells by using RNeasy Micro (Qiagen) following manufacturer’s instructions. The quality of RNA was assessed with a 2100 Bioanalyzer (Agilent Technologies). Cy3-labeled cRNA was prepared using a Low Input Quick Amp Labeling Kit according to the manufacturer’s protocol (Agilent Technologies). The labeled samples were hybridized to the Mouse Gene Expression v2 Microarray Slides (G4846A; Agilent Technologies) for 17 h at 65 °C at 10 rpm in a rotating hybridization oven. The Slides were washed and scanned with SureScan Microarray Scanner (Agilent Technologies) using a scan resolution of 5 μm and dye channel of Green with PMT set to 100%. Data was obtained using the Agilent Feature Extraction software (Agilent Technologies) with set to default for all parameters. The normalized expression data files were imported in GeneSpring GX12.6 software (Agilent Technologies) and 27,732 entities with values filtered by percentile (upper and lower cutoff; 100 and 20) and by flags (“detected” or “compromised” in at least two of all samples) were selected and shown in a scatter plot in Figure 2*A*. Two independent experiments were performed for transcriptome analysis. Data not presented in this study are available upon requests.

### Quantification and correlation analyses of image data

*Zsc4-EmGFP* Tg cells transiently transfected with the full-length Rif1 or its truncated mutants were fixed at 48 h and were subjected to immunostaining using anti-FLAG and GFP antibodies and costained with DAPI. Images were acquired by LSM780 (Zeiss) and over 400 cells in three representative images per each transformant were used for correlation analysis. For segmentation and quantification of three channels of signals of each cell, LSM format files were processed by MetaXpress version 5.1 (Molecular Devices) using the custom macro. Briefly, DAPI-stained nuclear areas were firstly defined, followed by segmentation of FLAG-stained nuclei and GFP-stained cytosol areas, and integrated signal intensities of binarized areas were assigned to DAPI total signals. Then FLAG and GFP signals in individual double-positive (FLAG(+) and GFP(+)) cells were analyzed for Spearman’s rank-correlation coefficients (rho), and corresponding two-tailed probability (*p*-value) was shown for each transformant: full-length, C552, or ΔN984 Rif1. Scattered plots were depicted by Excel.

### IVF, embryo collection and immunostaining

IVF of ICR mice was carried out as previously described elsewhere (79). Briefly, 8-week-old female ICR mice were super-ovulated by injecting 7.5 IU of pregnant mare serum gonadotropin (PMSG; ASKA Animal Health Co) followed by injecting 7.5 IU of human chorionic gonadotropin (hCG; ASKA Pharmaceutical Co) per body after 48 h. Oocytes were collected from the oviduct and transferred into HTF (ARK Resource) drops covered by mineral oil (Sigma-Aldrich) at 16 h post hCG injection. Fresh sperm were obtained from male ICR mice and capacitated in HTF for 1 h at 37 °C. After 6 h of coincubation of the oocytes with the sperm, fertilized oocytes were transferred into 100 μl drops of KSOM (ARK Resource) and continued to culture at 37 °C, 5% CO_2_. Fertilized oocytes (at 16 h post fertilization; 16 hpf) and embryos at 2C (20, 30, or 40 hpf; only data at 40 hpf were shown in Fig. 8, *B* and *G*), four-cell (48 hpf), eight-cell (at 2.5 days post fertilization; 2.5 dpf), morula (3 dpf) and blastocyst (3.5 dpf) were collected and fixed with 4% paraformaldehyde in PBS for 15 min, washed with PBS three times, permeabilized with 0.2% Triton X-100/PBS for 15 min, and blocked with 5% normal goat serum/PBS for 1 h at RT. Embryos were incubated with primary antibodies overnight at 4 °C, washed, and incubated with anti-mouse Cy3 (1/1000 dilution; Jackson ImmunoResearch) and anti-rabbit Alexa Fluor 488 (Molecular Probes) for 1 h at RT. DNA was stained with 1 μg/ml DAPI for the following 10 min. The samples were once adapted to 40% glycerol and mounted with 90% glycerol/2.5% DABCO. For immunostaining of unfertilized eggs, collected samples were soaked with 0.15% hyaluronidase (Sigma-Aldrich) in KSOM for 15 min at 37 °C to remove cumulus cells.

IVF and immunostaining of C57BL/6J embryos were conducted as above, except for several modifications. In this case, embryos were incubated primarily with anti-Nanog antibody, followed by labeling with secondary Cy-3-conjugated anti-rabbit IgG, and tertiary Alexa Fluor 488-conjugated anti-Rif1N antibody. For analysis at 2C stage, B6 embryos at 24 hpf for G1 or S phase and at 40 hpf at G2 phase (80) were collected and used. To examine the effect of inhibition of nuclear export, embryos were incubated with 40 nM of leptomycinB (Sigma-Aldrich) for 4 h before harvest and Rif1 was detected with anti-Rif1N antibody. Images were acquired by LSM510 (Zeiss; Fig. S5*A*) and LSM780 (Zeiss; Figs. 8 and S5, *B* and *C*) with PlanApo63×/1.4 lens.

For immunoblot analysis of C57BL/6J mouse oocytes and embryos, IVF was conducted as above with minor modifications; 0.2 ml CARD HyperOva (Kyudo Co Ltd) per body in place of PMSG was injected into 4-week-old animals, and homemade HTF and KSOM were used. The unfertilized eggs were treated with 0.1% hyaluronidase for 1 min at 37 °C. Before collection, eggs and embryos were washed in custom-made KSOM BSA(−) medium (depleted of BSA and supplemented with protease inhibitors) and were transferred with a small volume of the same medium to the stock tubes.

### Mapping of RNA-seq reads on the mouse genome

Public total RNA-seq fastq data of MII eggs and embryos (at one-cell, 2C, four-cell, morula and blastocyst stages) was collected from ArrayExpress (#E-MTAB2950) (40), and mRNA-seq of mouse ES cells was from Gene Expression Omnibus data repository (GEO; GSE93453) (81). After processing by Trimmomatic software (version 0.39) (82), filtered paired-end reads were mapped onto mm10/NCBI38 mouse reference genome using by HISAT2 (version 2.2.1) (83). BAM files were loaded on IGV genome browser (84) and presented.

### Statistics

In most of this study, statistical significance of two sets of data was analyzed by two-tailed Student’s *t* test and shown by asterisk marks explained in the figure legends or shown with *p*-values. For qRT-PCR and ChIP-qPCR, the means of technical triplicates with ±SD were shown. For quantification and correlation, analyses of image data were described elsewhere.

## Data availability

The nucleotide sequence of mouse Rif1 cDNA originated from 129/Sv strain is available at GenBank with accession number JX255737. Microarray data are available in GEO with accession number GSE158220.

## Supporting information

This article contains supporting information (27, 85, 86, 87, 88, 89, 90).

## Conflict of interest

The authors declare no conflicts of interest in regard to this manuscript.
