# Supplementary material for: Loss of full-length DNA replication regulator Rif1 in two-cell embryos is associated with zygotic transcriptional activation
Source: J Biol Chem. 2021 Nov 1;297(6):101367. doi: 10.1016/j.jbc.2021.101367 (PMC8686075; doi:10.1016/j.jbc.2021.101367)
Supplement: Figures S1–S6 [file mmc1.pdf]

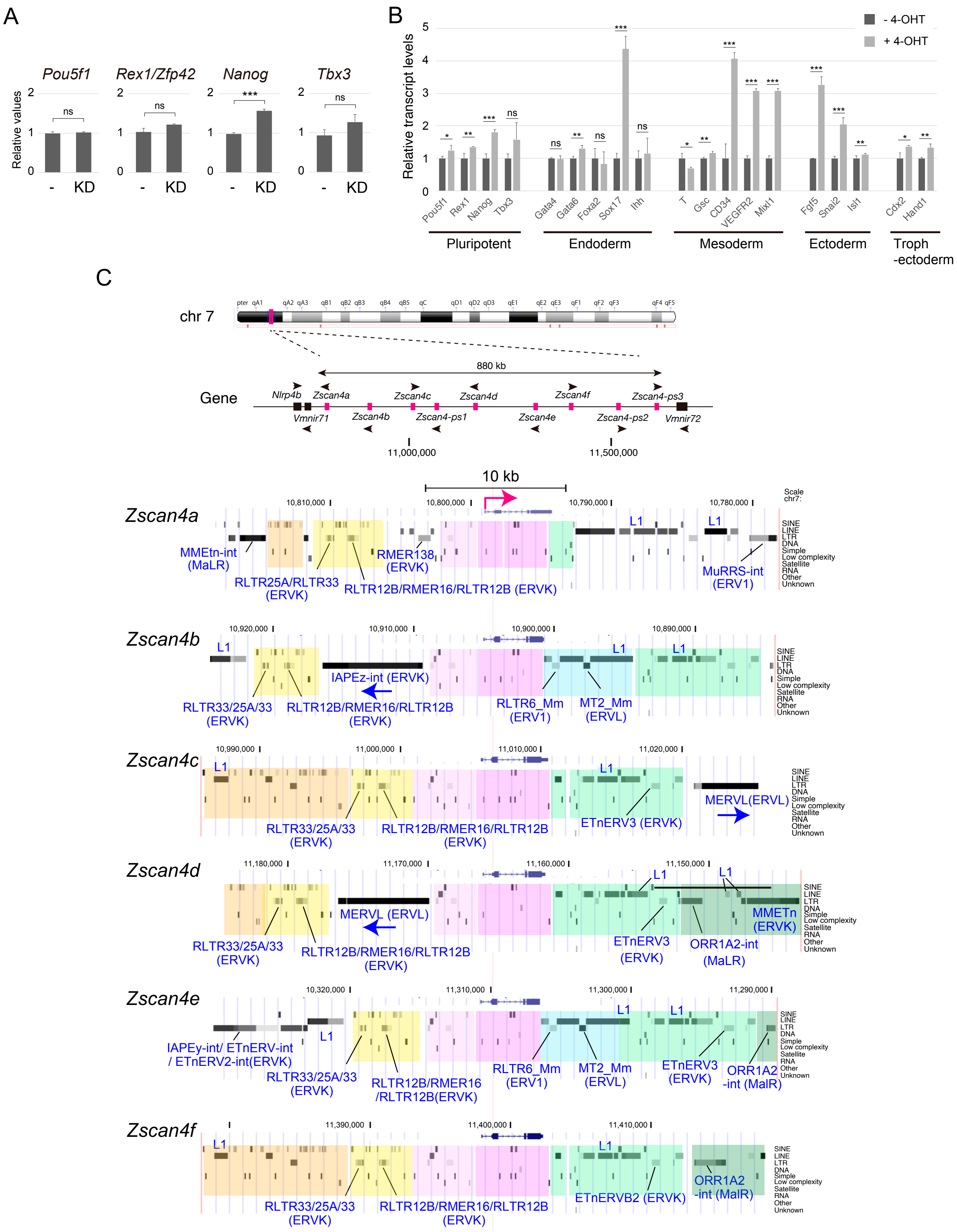

Figure S1. Effect of Rif1 depletion on the expression of various transcription factors in mouse ES cells.
A. qRT-PCR analysis of pluripotent markers in Rif1 knockdown cells. E14tg2a cells transfected twice with siRNA Rif1#2 were harvested at 72 h. Relative values (KD) to control knockdown (-, set as 1) are shown.
B. qRT-PCR analysis of pluripotent and differentiation markers in Rif1 knockout cells. *Rif1$^{f/f}$ CreERT2* cells treated with 4-OHT or mock treated were cultured for 48 h and the relative transcript levels of indicated genes are shown. In A and B, two independent experiments were performed and the means of the technical triplicates in the representative data are plotted. Bars, SDs. *, *P*<0.05. **, *P*<0.01. ***, *P*<0.001. ns, not signifcant.
C. Genomic DNA structure of the *Zscan4* gene loci. Map of the *Zscan4* family genes (*a* to *f*) and surrounding repetitive elements derived from UCSC Genome Browser (NCBI38/mm10) is shown. The conserved homologous regions are color-coded. Black or gray bars, repeats; arrows, direction of *Zscan4* (magenta) or ERV (blue) transcripts.

A

Signal intensity of GFP(+) population

Zsc4-EmGFP Tg cells

	Fluorescence (A.U.)
Control siRNA	6611 ± 625
Rif1 siRNA	12422 ± 475
Fold change	1.88 ± 0.072

** (Control siRNA vs Rif1 siRNA)

Zsc4-EmGFP KI cells

	Fluorescence (A.U.)
Control siRNA	5019 ± 401
Rif1 siRNA	6772 ± 144
Fold change	1.35 ± 0.029

** (Control siRNA vs Rif1 siRNA)

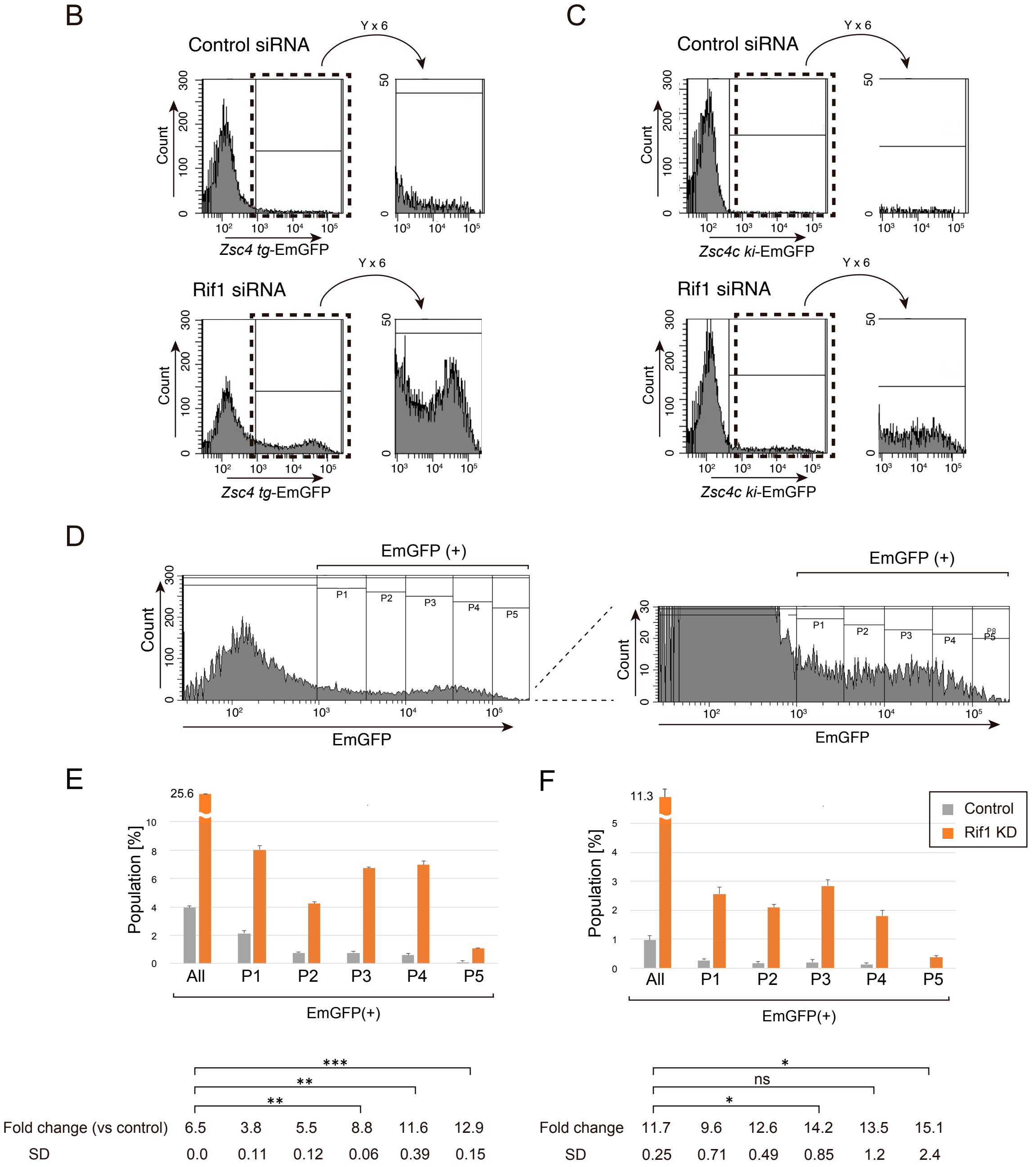

Figure S2. (Related to Figure 2F) The fluorescent signal intensity levels of Zscan4-EmGFP increase by Rif1 depletion.

A. The signal intensities of Zscan4-EmGFP in GFP(+) populations. B and C. Histograms of EmGFP in control (upper panels) or Rif1-knockdown cells (lower panels) using *Zsc4-EmGFP* Tg (B) or *Zsc4-EmGFP* KI (C) cells. D to F. EmGFP-positive populations were divided into 5 fractions according to the fluorescence intensity. In D, a representative histogram, expanded on x-axis, are shown. E and F. The GFP-positive population (%; top) and its fold change (Rif1 knockdown versus control; bottom) of each fraction are shown. The means of biological triplicates are shown with SD. The fold change values were statistically evaluated by two-tailed student' s *t*-test and those with significantly higher fold change than in total population were indicated. *, $P<0.05$. **, $P<0.01$. ***, $P<0.001$. ns, no significance. no marks, significantly lower ($P<0.05$). Three independent experiments were performed and the representative histograms are shown in B to D.

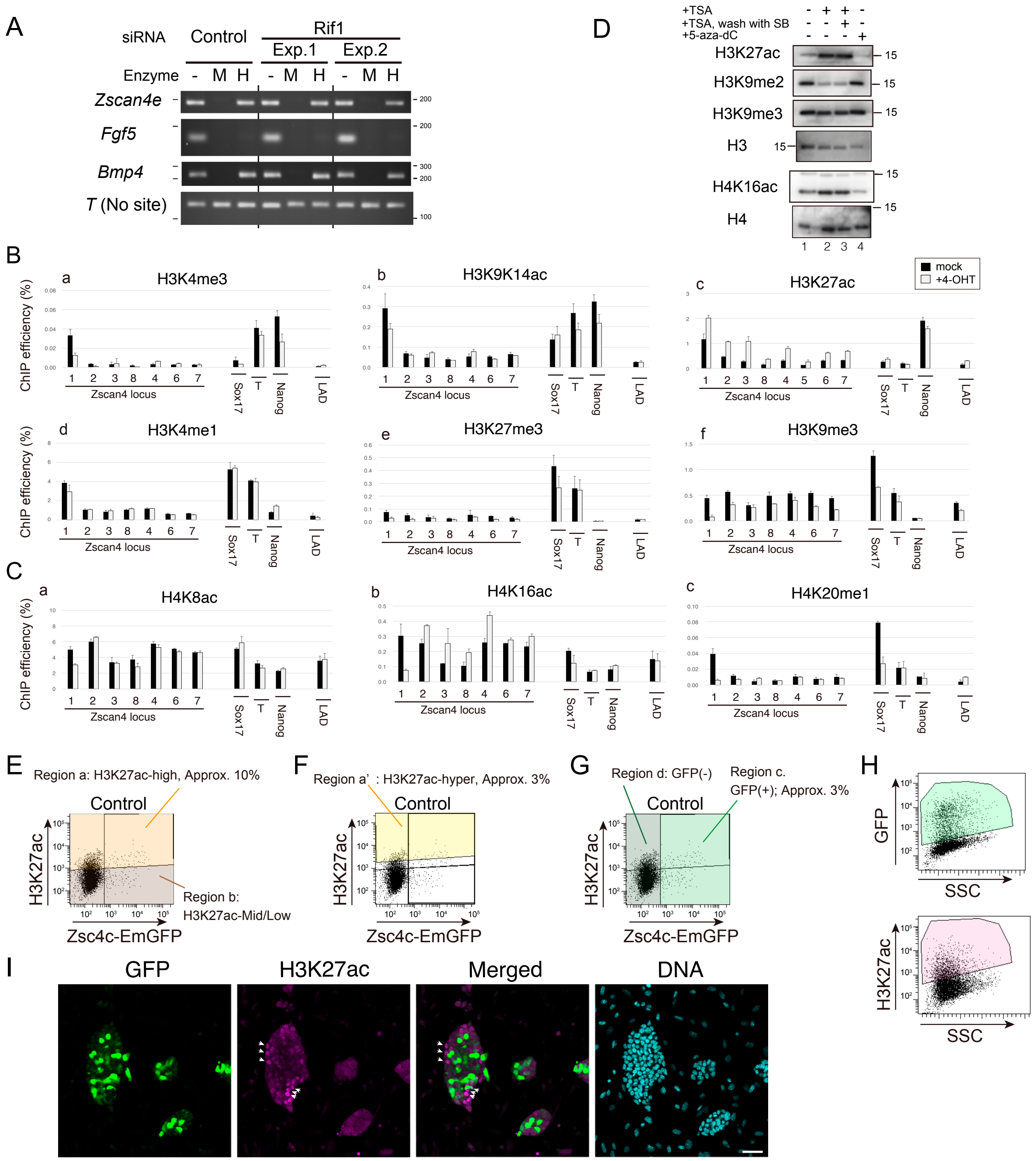

Figure S3. (Related to Figure 3 and 5) DNA methylation and histone modification analysis of Rif1-depleted cells
A. DNA methylation analysis by HpaII assay. Genomic DNA was prepared from Rif1 siRNA-treated or untreated E14tg2a cells and digested by HpaII, MspI or mock treated. *Fgf5* (amplicon in exon 1) and *Bmp4* (exon 4) were analyzed as controls for unmethylated and methylated loci, respectively. *T* (230 bp upstream of *T* gene), containing no MspI site, was analyzed as an uncleavable control. B and C. ChIP analysis of Rif1 null cells. *Rif1*$^{f/f}$ *CreERT2* cells treated with 4-OHT or mock treated were cultured for 58 h and subjected to ChIP assay. The results of ChIP-qPCR analysis of histone H3 modification (B) or histone H4 modification (C) were shown. The target regions of *Zscan4* ChIP primers are shown in Fig. 3D. D. Immunoblots of histone modification in control samples. E14tg2a cells were treated with tricostatin A (5 µM, 16 h; lanes 2 and 3), 5-aza-deoxycytidine (5 µM, 48 h; lane 4) or untreated (lane 1). At the harvest, cells were washed with PBS containing sodium butylate (SB) for lane 3. The histone-enriched fractions were prepared by acid extraction methods as in Experimental procedures, and 0.1 µg or 3 µg of proteins were used for detection for pan H3/H4 or modified histones, respectively. Bars, 15 kD markers. E. (related to Figure 5D and 5F) Selected regions for H3K27ac-high (region a; approximate 10% in untreated cells) and H3K27ac-mid/low (region b). F. (related to Figure 5E) The selected region for H3K27ac-hyper population. G. (related to Figure 5D and 5F) Selected regions for GFP -positive (region c; 3% in untreated cells) or GFP-negative population (region d). In E to G, five gate definitions were shown in the identical replicate. H. (related to Figure 5G and 5H) GFP-positive (top, green) and H3K27ac-high (bottom, magenta) fractions are shown. I. Zscan4-positive events are not completely concurrent with high H3K27 acetylation. *Zscan4c-EmGFP* KI cells were transfected with control or mRif1#2 siRNA twice and subjected to immunofluorescence microscopy using indicated antibodies. Only mRif1 siRNA-treated cells were shown. Arrowheads, H3K27ac-highly stained cells without GFP signals. Bars, 50 µm.

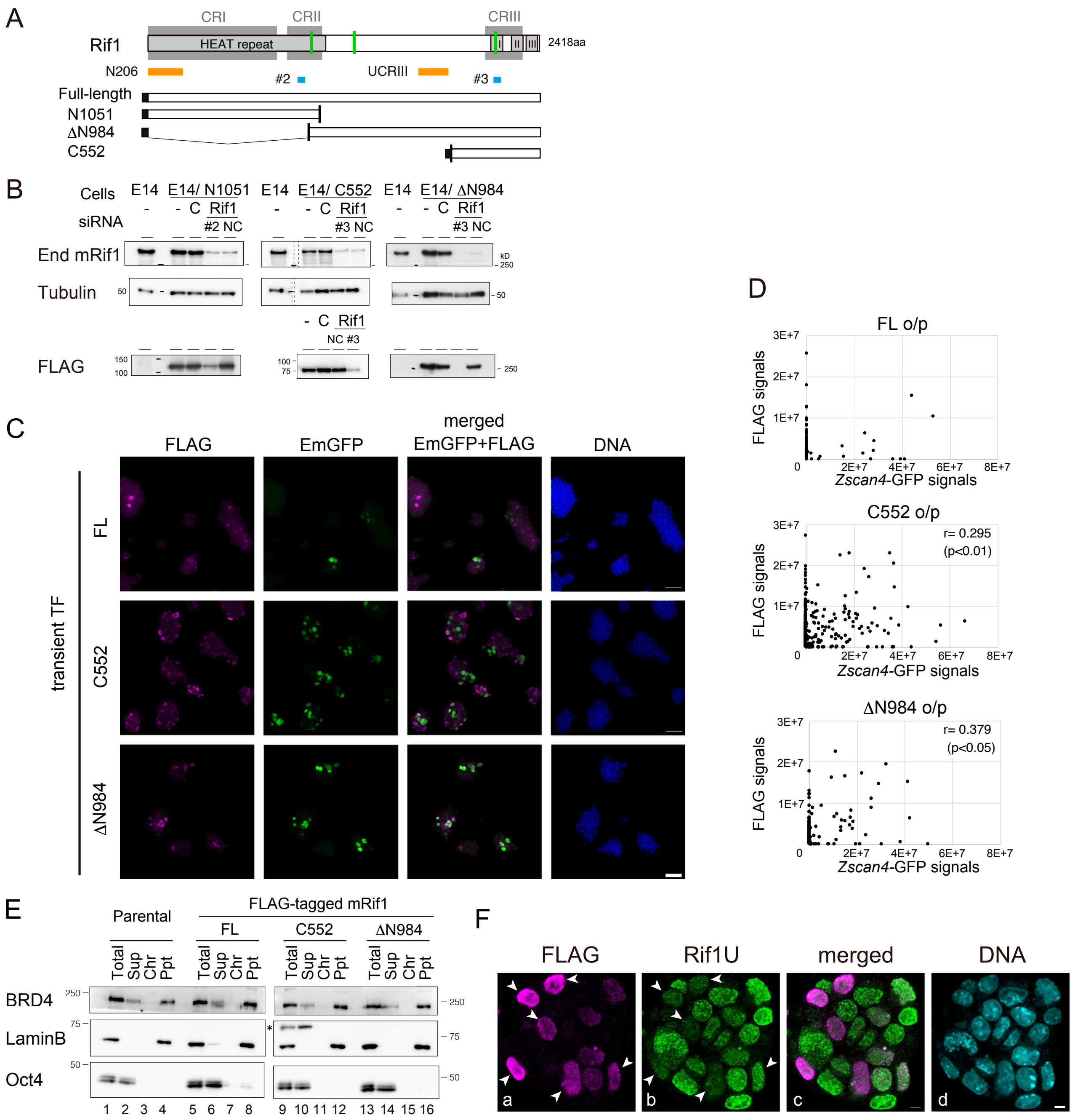

Figure S4. Analysis of Rif1 truncated mutants.

A and B. (Related to Figure 6C) Immunoblots for the complementation assays. In A, a schematic of deletion variants is shown (same as in Figure 6A). In B, immunoblot analysis. The E14tg2a stable transformants expressing FLAG-Rif1 N1051, C552 or ΔN984 were transfected with Rif1 siRNA #2, #3, NC#1 or control siRNA (C) twice and harvested at 48 h. The whole cell extracts (10 µg) were analyzed for the expression of truncated Rif1 mutants by immunoblot using anti-FLAG antibody. Endogenous Rif1 was detected with anti-Rif1 N206 (Rif1N) for N1051 mutant and with anti-Rif1 UCRIII (Rif1U) for C552 and ΔN984 mutants. -, untreated. The locations of siRNA targets are indicated in A (blue). siRNA NC#1 targets 3’ UTR of Rif1. C and D. Correlation between the levels of overexpressed FLAG -Rif1 truncated proteins and those of Zscan4-EmGFP signals in single cells. In C, representative images of immuno -staining of *Zscan4-EmGFP* Tg cells transiently transfected with full-length (FL), C552 or ΔN984 for 48 h with anti-FLAG and GFP antibodies. Bar, 50 µm. In D, FLAG and GFP signals of individual cells were plotted and the correlation coefficients (r; *rho*) were calculated by the Spearman’ s rank correlation method. The *P*-values of double -positive cells are presented. Correlation analysis was not applied to full-length Rif1-expressing cells since the number of double-positive cell population was too small (11 cells). Total 514, 759 or 425 cells for FL, C552 or ΔN984 were analyzed, respectively. E. (Related to Figure 7B) Immunoblot analysis of the cellular fractionations of ES cells transiently transfected with FLAG-tagged full-length or truncated Rif1 mutants. The whole cell lysates (10µg; Total), the cytoplasmic and nuclear soluble fractions (Sup), the chromatin fractions (Chr), and the nuclear scaffold-enriched fractions (Ppt), were subjected to immunoblot and detected with indicated antibodies. An asterisk, non-specific signals. F. Nuclear Rif1 signals detected with anti-Rif1U antibody also decreased in Rif1 C552-overexpressing cells. E14tg2a cells transiently transfected with FLAG-Rif1 C552 were fixed at 48 h and immuno-stained with indicated antibodies. Representative confocal microscopic images are shown. A minumum of two independent experiments were performed for each analysis. Bar, 5 µm.

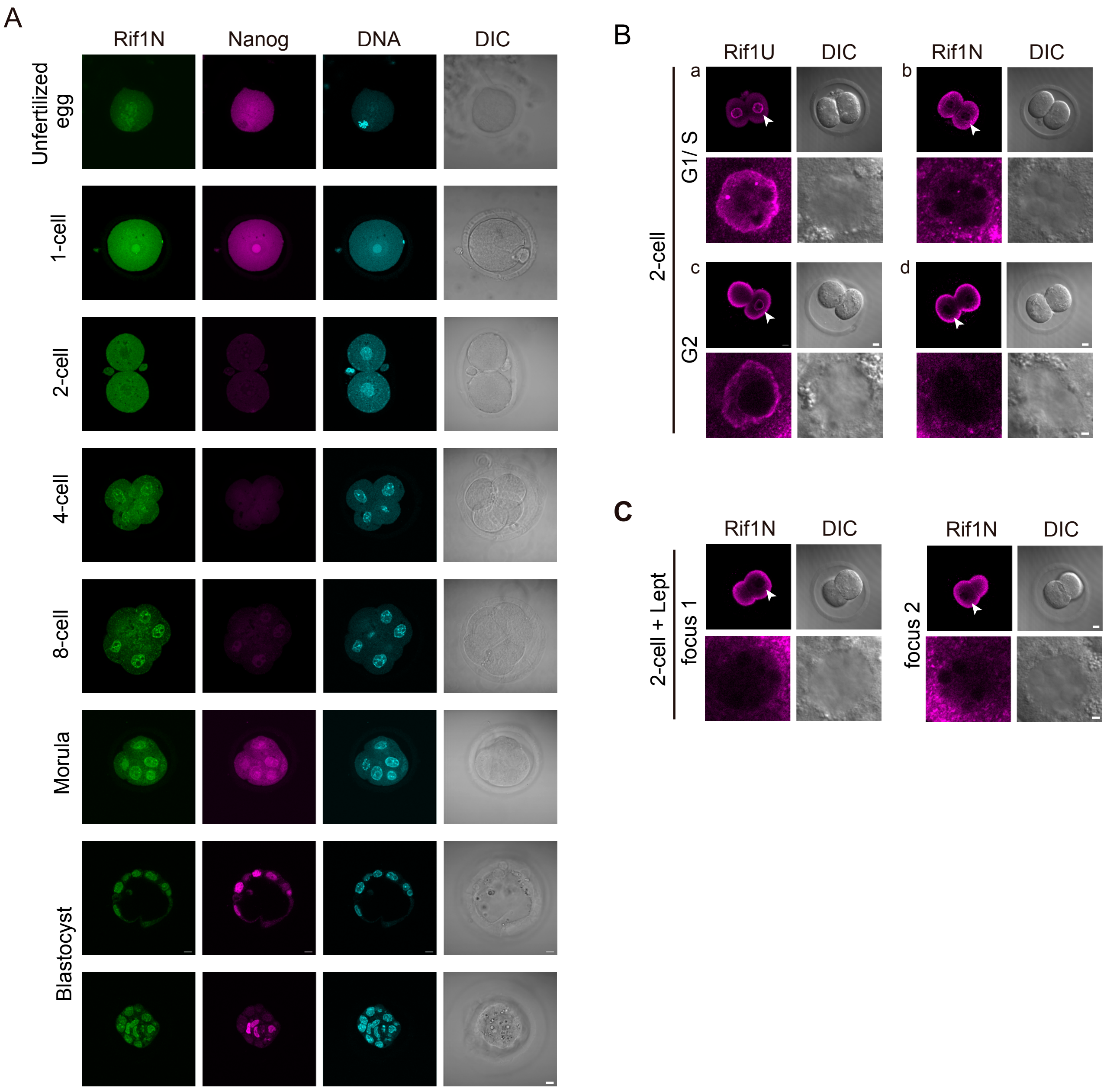

Figure S5. Cellular localization of Rif1 in preimplantation embryos (C57BL/6J mice)
A. Whole-mount immunostaining of C57BL/6J mouse MII oocytes (top panels) and embryos during preimplantation development using anti-Rif1N antibody and anti-Nanog antibody. DNA was costained with DAPI. Bar, 10 µm. B. Two-cell embryos at early stage (a and b) or late stage (c and d) were fixed and subjected to immunostaining with anti-Rif1N or Rif1U antibodies. C. Two-cell embryos were incubated with leptomycin B, fixed, and Rif1 was detected with anti-Rif1N antibody. In B and C, each panel set shows original images (upper panels) and enlarged images acquired by digital zoom at × 6 (lower panels). In B and C, bars, 10 µm (upper panels) or 2 µm (lower panels). The representative data of one out of two independent experiments were shown.

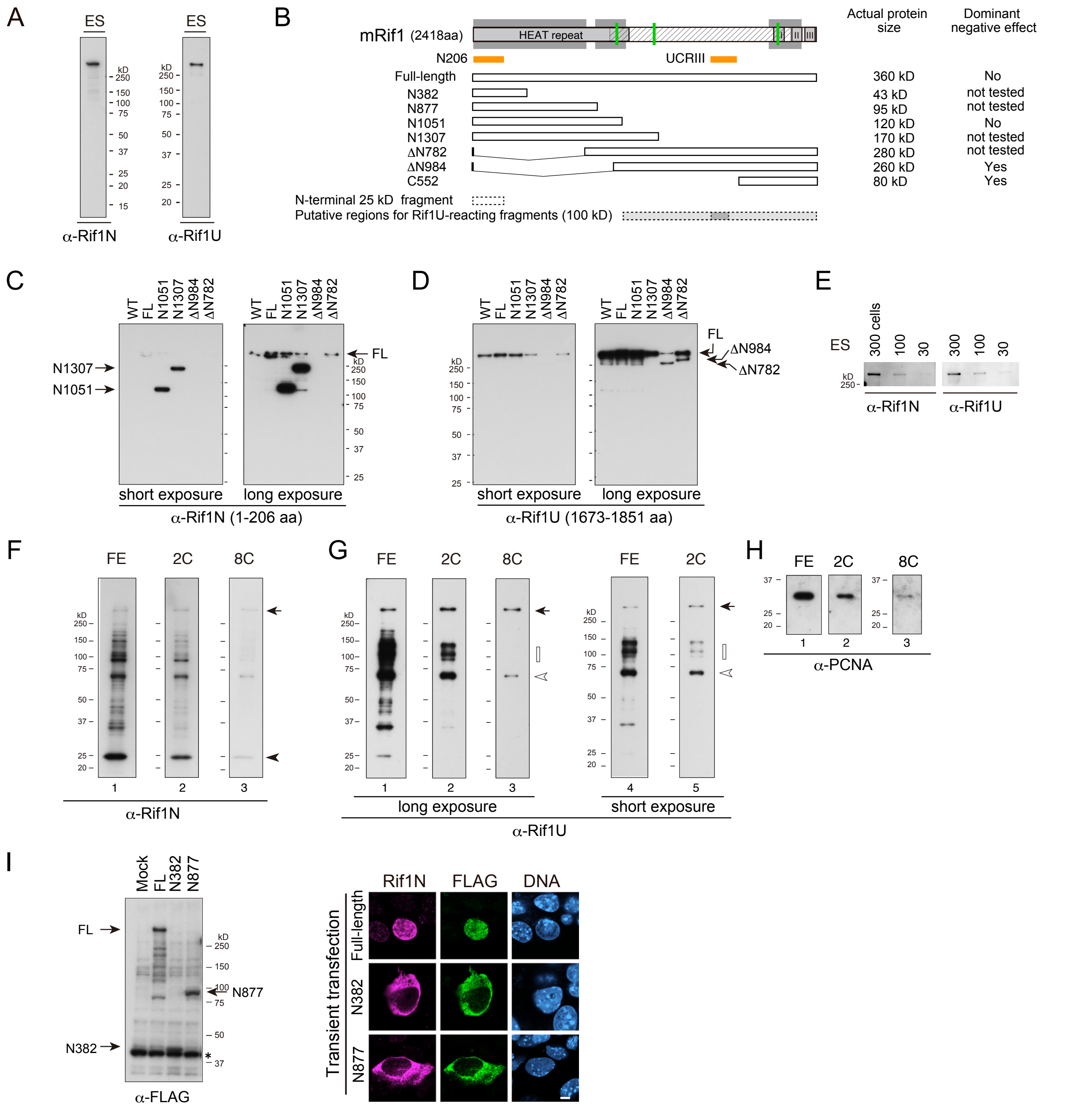

Figure S6. Rif1 expression in fertilized eggs, 2-cell and 8-cell embryos (Related to Figure 9)

A. Validation of anti-Rif1 antibodies. The whole cell extracts (10 µg proteins) of E14tg2a cells were subjected to immunoblot analysis by using affinity-purified anti-Rif1 N206 (Rif1N) or anti-Rif1 UCRIII (Rif1U). B. A schematic drawing of the full-length and truncated Rif1. Green bars, NLS. Orange bars, the antigens for Rif1 antibodies. A shaded column, IDR. Gray boxes, conserved regions. Estimated locations of the N-terminal 25 kDa polypeptide and C-terminal polypeptides are indicated by dotted boxes. C and D. Validation of anti-Rif1 antibodies. *Zscan4 -EmGFP* MC1 mouse ES cells were transfected with vectors expressing mRif1 full-length or 4 truncated forms, and the whole cell extracts (10 µg proteins) were analyzed by immunoblot by using anti-Rif1N (C) or anti-Rif1U (D) antibody. E. Detection of endogenous Rif1 in small numbers of E14tg2a mouse ES cells by anti-Rif1 antibodies. F, G, and H. Immunoblot analysis of fertilized eggs at 6 hpf (FE), embryos at 2-cell (2C) or 8-cell (8C) stage. The whole cell extracts derived from 150 cells were analyzed by using anti-Rif1N (F), anti-Rif1U (G) or anti-Pcna (H). For fertilized eggs and 2-cell embryos in G, short exposure data is also shown. The possible N-terminal 25 kD is indicated by closed arrowheads, and the potential C-terminal 67 kD or larger fragments, by open arrowheads or open rectangles, respectively. Arrows, full-length Rif1. In H, the expression levels of Pcna per cell decreased during development, whereas those per embryo are almost constant (in Figure 9C c). This is probably because the maternal Pcna is diluted during cell expansion and the zygotic expression level is much lower than that in oocytes. I. Cellular localization of N-terminal fragments of Rif1. E14tg2a cells were transiently transfected with DNA expressing FLAG-tagged full-length, N382 or N877 Rif1 for 48 h. The whole cell extracts (20 µg proteins) were analyzed by immunoblot with anti-FLAG antibody (left). Astarisk, non-specific bands. Cellular localization of full-length, N382 or N877 was examined by immunostaining with anti-Rif1N (magenta) or anti-FLAG (green) antibody (right). DNA was costained with DAPI (blue). Bar, 5 µm. A minimum of two independent experiments were performed for each analysis.
